# Exploring the bee fauna on the islands of Brittany (France): an initial survey reveals a remarkable species richness

**DOI:** 10.3897/BDJ.13.e138570

**Published:** 2025-02-14

**Authors:** Violette Le Féon, Mael Garrin, David Genoud, Matthieu Aubert, Éric Dufrêne, Marie Filipe, Floriane Flacher, Thibault Ramage, Anthony Stoquert, Stéphane Vassel, Benoît Geslin

**Affiliations:** 1 Independent, 35590 Saint-Gilles, France Independent 35590 Saint-Gilles France; 2 Groupe d’Étude des Invertébrés Armoricains, 104 rue Eugène Pottier, 35000 Rennes, France Groupe d’Étude des Invertébrés Armoricains, 104 rue Eugène Pottier 35000 Rennes France; 3 Independent, 87240 Ambazac, France Independent 87240 Ambazac France; 4 Independent, 34380 Pégairolles-de-Buèges, France Independent 34380 Pégairolles-de-Buèges France; 5 Observatoire des Abeilles, 30170 Saint-Hippolyte-du-Fort, France Observatoire des Abeilles 30170 Saint-Hippolyte-du-Fort France; 6 NaturaGIS, 44520 Moisdon-la-Rivière, France NaturaGIS 44520 Moisdon-la-Rivière France; 7 Correspondant du Muséum national d'Histoire naturelle de Paris, 29900 Concarneau, France Correspondant du Muséum national d'Histoire naturelle de Paris 29900 Concarneau France; 8 Independent, 56400 Plumergat, France Independent 56400 Plumergat France; 9 Independent, 23300 Saint-Agnant-de-Versillat, France Independent 23300 Saint-Agnant-de-Versillat France; 10 Université de Rennes (UNIR), UMR 6553 ECOBIO, CNRS, 263 avenue du Général Leclerc, 35042 Rennes cedex, France Université de Rennes (UNIR), UMR 6553 ECOBIO, CNRS, 263 avenue du Général Leclerc 35042 Rennes cedex France

**Keywords:** conservation, geographical distribution, heathland, Hymenoptera Apoidea Anthophila, insular biodiversity, pollinator insects, sandy habitats, thermophilous species, threatened species

## Abstract

Islands are areas where biodiversity conservation is of the utmost importance and is particularly challenging due to the isolation and vulnerability of animal and plant populations. The coastline of Brittany includes a large number of islands, which vary greatly in size, distance from the mainland, landscape composition and climate. Until recently, virtually nothing was known about the bees on these islands, but a number of studies have been carried out in recent years, allowing an initial assessment to be made. The aim of this article is to provide an overview of the bee fauna of the islands of Brittany, in terms of species richness, species composition and rarity status. In total, we gathered records of 188 wild bee species on 25 continental islands, located on both the north and south coasts of Brittany. For most of the islands, we obtained only occasional data, but a few have benefitted from intensive surveys, with data collected throughout the entire flight period and over several years and in different locations and habitat types. For four islands, we considered that the current knowledge is relatively good: Groix (113 wild bee species), Houat (82 species), Hoedic (64 species) and Ouessant (57 species). In addition to the number of species, this study shows that the islands host many species that are rare at regional or national level. Our results highlight the importance of taking bees into account when managing habitats and defining protected areas in islands, in order to conserve both food resources and nesting sites for these pollinator insects.

## Introduction

Pollinators are experiencing an unprecedented decline due to increasing human pressures on ecosystems (Powney et al. 2019, Dicks et al. 2021). In this context, gathering data on their geographical distribution and status is the first step towards implementing appropriate conservation measures (Brown and Paxton 2009, Nieto et al. 2014, Reverté et al. 2023, Marshall et al. 2024). Islands are areas where biodiversity conservation is of the utmost importance and is particularly challenging due to the isolation and vulnerability of animal and plant populations (Blondel 1995, Whittaker and Fernández-Palacios 2007, Kier et al. 2009, Geslin et al. 2023). In Europe, the bee fauna of some archipelagos, such as the Balearic Islands (Baldock et al. 2020, Owens and Riddiford 2022), the Maltese Islands (Balzan et al. 2016, Balzan et al. 2023), the Channel Islands (Richards 1978, Beavis 1999, Else and Edwards 2018) and the Isles of Scilly (Beavis 2013, Beavis 2024) have been studied for a long time.

In France, in general, there is still a lot to learn about the distribution of bees (Schatz et al. 2021). Regarding the islands in metropolitan France, a few publications exist for Mediterranean islands (e.g. Coiffait-Gombault et al. (2016) for Porquerolles and Le Divelec et al. (2024) for Corsica), but knowledge is still very incomplete. Regarding the overseas islands, recent publications provide checklists of the bees of New Caledonia (Zakardjian et al. 2023) and Guadeloupe (Le Coeur et al. 2023). In metropolitan France, Brittany, in the north-west of the country, is the region with the largest number of islands (around 70% of the islands in metropolitan France, according to the Observatoire de l'Environnement en Bretagne (2018)). These islands have long played a major role in the conservation of flora (e.g. Bioret and Malengreau (2001), Bioret (2002), Rivière (2007), Laurent et al. (2022)), seabirds (GOB 2012, Cadiou et al. 2023) and marine mammals, such as the grey seal *Halichoerusgrypus* (Vincent et al. 2005). They are also important areas for several species of invertebrates, both coastal species (e.g., *Nebriacomplanata*, Tiberghien (2004), Ramage (2019)) and species not specifically associated with coastal habitats (e.g. several butterfly species, Buord et al. (2017), Garrin (2018), Garrin (2020)).

Until recently, there were virtually no data on the bee fauna on the islands of Brittany. In recent years, however, a number of studies have been carried out, providing the first insights into these bee communities (e.g. Garrin 2018, Le Féon et al. 2018, Garrin 2019, Garrin 2020, Le Féon 2023). The aim of this article is to present the data currently available and to provide a first assessment of the bee fauna composition on the islands of Brittany. We will examine species richness, species composition, rarity status and ecological requirements of the species.

## Material and methods

### Study area

Our study focused on Brittany, an administrative region in the north-west of France, bordered by the English Channel to the north, the North Atlantic to the west and the Bay of Biscay to the south. Due to its geographical position and geological structure, Brittany includes a very large number of continental islands: nearly 800 according to Brigand and Bioret (2002), representing more than 15000 ha in total. They are distributed unevenly around the coastline of the four Breton departments (Côtes d'Armor, Finistère, Ille-et-Vilaine and Morbihan), but they are particularly numerous off the coasts of Finistère, Morbihan and the west of the Côtes d'Armor. These islands vary greatly in size: 75% are smaller than 1 ha, 10% are between 1 and 5 ha and only 15% are larger than 5 ha (Brigand and Bioret 2002). Ranked in descending order of surface area, the main inhabited islands are: Belle-Île-en-Mer (8563 ha), Ouessant (1558 ha), Groix (1482 ha), Batz (370 ha), Arz (330 ha), l'île aux Moines - in the Gulf of Morbihan (320 ha), Bréhat (309 ha), Houat (288 ha), Hoedic (209 ha), Molène (75 ha), Sein (58 ha) and Saint-Nicolas des Glénan (35 ha).

Brittany has a temperate oceanic climate, so it is characterised by mild, rainy winters and cool, relatively wet summers. Temperature, rainfall and sunshine are not evenly distributed (Buord et al. 2017) and there is a gradient of increasing temperature and sunshine duration from the north-west to the south-east of the region. The islands of Belle-Île-en-Mer, Hoedic and Houat, in the south-east of Morbihan, are amongst the warmest and sunniest places in the region.

Thanks to their isolation and protection measures (many of them benefit from protection such as the European Natura 2000 network, national or regional nature reserves, regional nature parks and marine nature park), the islands of Brittany are home to a wide variety of well-preserved natural and semi-natural habitats. Globally, the most common habitats are dunes, grasslands, heaths and thickets (see Suppl. material 1 that contains a vegetation map, based on Sellin et al. (2021), for islands considered in our study). The high floristic value of grasslands has been highlighted, in particular in Belle-Île-en-Mer (e.g. Laurent et al. (2022)). On some of the islands (Batz, Belle-Île-en-Mer and Groix), arable lands are relatively well represented, but farming practices often comply with organic or extensive farming principles. Finally, in the studied islands, the towns and villages often have large areas of flower-rich gardens.

**Figure 1.**
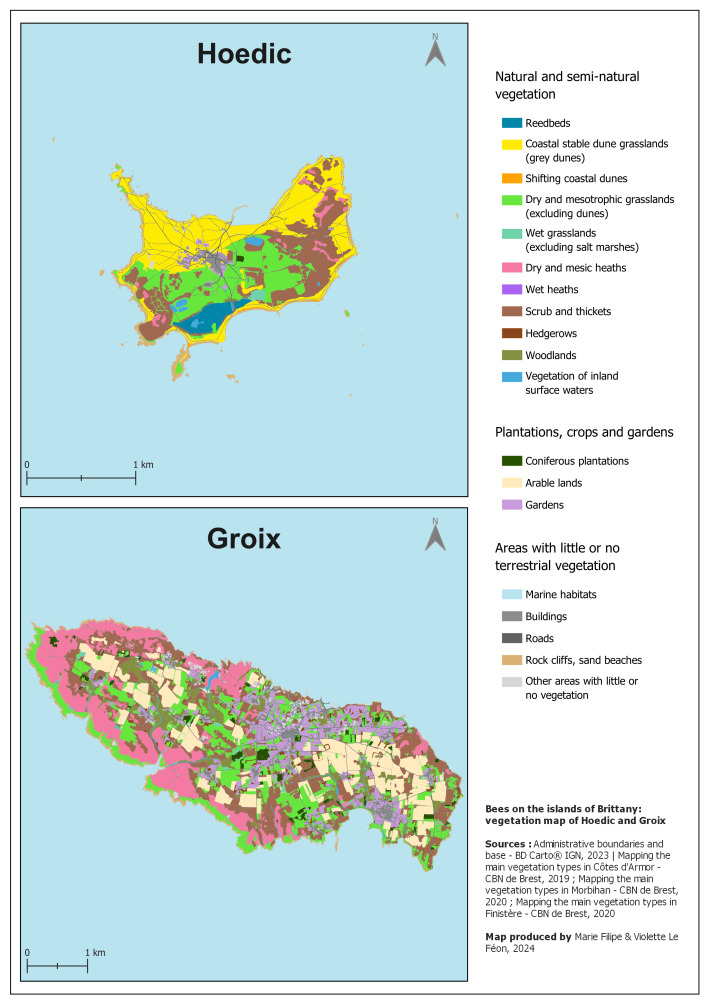
Vegetation map of the islands of Hoedic and Groix, based on Sellin et al. (2021). This figure shows two contrasting islands in terms of landscape composition. Grey dunes occupy large areas on Hoedic, whereas they are absent on Groix. In constrast, heathland is very common on Groix, but rare on Hoedic. Groix also has large areas of arable land (absent on Hoedic) and gardens (smaller area on Hoedic). Grassland and scrubland are present on both islands.

The proportions of the different habitat types vary greatly from one island to another. On some islands, such as Houat, Hoedic, Saint-Nicolas des Glénan and Aganton, dunes occupy large areas, whereas they are absent or almost absent from other islands. Similarly, the proportion of the surface area covered by heathland varies greatly. Ouessant and Groix, for example, are islands where heathland occupies large areas (see Fig. 1, which shows two contrasting islands in terms of landscape composition: Hoedic and Groix).

### Data collection

Our data collection focused on wild bee species, i.e. all bee species (Hymenoptera Apoidea Anthophila) with the exception of the honey bee (*Apismellifera*). Hereafter, the term bees is used for wild bees. We are aware that *Apismellifera* is present on several islands, either due to beekeeping activities or as wild populations (e.g. on Groix). On islands such as Belle-Île-en-Mer, Ouessant and Groix, efforts are being made to conserve the subspecies *Apismelliferamellifera*. However, we have chosen not to include *Apismellifera* in our study because field observations by naturalists often do not give this species the same attention as wild bees. This species is sometimes under-reported or omitted because it is considered ubiquitous or not part of the wild fauna. Including *Apismellifera* under the same criteria as other bee species could have led to biased results in our analyses and we, therefore, decided to remove it from the list.

We contacted entomologists and organisations likely to have bee occurrence data on the islands of Brittany. We collected data from the databases of the GRETIA (Groupe d’Étude des Invertébrés Armoricains, http://www.gretia.org/), the main organisation dedicated to entomology in Brittany and from the Observatoire des Abeilles (https://oabeilles.net/), a national association dedicated to the study of bees in France. Other data were collected directly from entomologists. All data came from specimens identified by specialists in bee taxonomy (either co-authors of the present paper or listed in the Acknowledgement section). The taxonomic reference system used is TAXREF version 17.0 (TAXREF 2024). In some cases, the data have been included in a study report or an article. The corresponding references are given in Table 1. These references provided useful metadata about the context of the study and the sampling method and effort.

We looked up information on the status of the species and their geographical distribution in the following references: Nieto et al. (2014), Observatoire des Abeilles (2018), Hubert et al. (2023). In the absence of a French Red List of bees (Schatz et al. 2021), the IUCN European Red List of bees (Nieto et al. 2014) is the reference document in the geographical area covered by our study. We consulted the departmental checklists of bees in the Brittany region and nearby departments (Armorican Massif area) to find out the known distribution of species in these departments (based on current knowledge and sometimes probably incomplete). For the departments in Brittany (Côtes d'Armor, Finistère, Ille-et-Vilaine and Morbihan) and for the departments in Normandy (Calvados, Manche, and Orne), the lists dated from 2018 (Observatoire des Abeilles 2018). For the departments in the Pays de la Loire region (Loire Atlantique, Maine-et-Loire, Mayenne, Sarthe and Vendée), there was a recent update (Hubert et al. 2023). We supplemented our search for information about bee ecology and distribution with relevant national and European references, such as: Bogusch and Straka (2012), Falk and Lewington (2015), Pauly and Belval (2017), Else and Edwards (2018), Michez et al. (2019), Westrich (2019) and Rasmont et al. (2021).

### Assessment of the level of knowledge

We considered three groups of level of knowledge, based on the number of occurrence records we obtained (defined as the observation of a number of specimens of a given taxon at a given location on a given date):

- low level of knowledge: less than 10 occurrence records;

- medium level of knowledge: between 10 and 100 occurrence records;

- good level of knowledge: more than 100 occurrence records.

## Results

### Geographical and temporal coverage

We collected 1636 occurrence records. Each row of the database, provided in Suppl. material 2, represents an occurrence record, defined as the observation of a number of specimens of a given taxon at a given location on a given date. We tried to obtain the most accurate data possible, i.e. respectively, the number of specimens, the species, the geographical coordinates and the exact day of the year. However, sometimes only approximate information was available, i.e. an approximate number of specimens, an identification to the genus level or to the group of species, the year and the island without any accurate location. Most of the observations come from opportunistic data, the bees having been caught with a net. In some cases, bees were caught with coloured pan traps, Malaise traps and Barber pitfall traps (see the column "sampling method" in Suppl. material 2).

**Figure 2.**
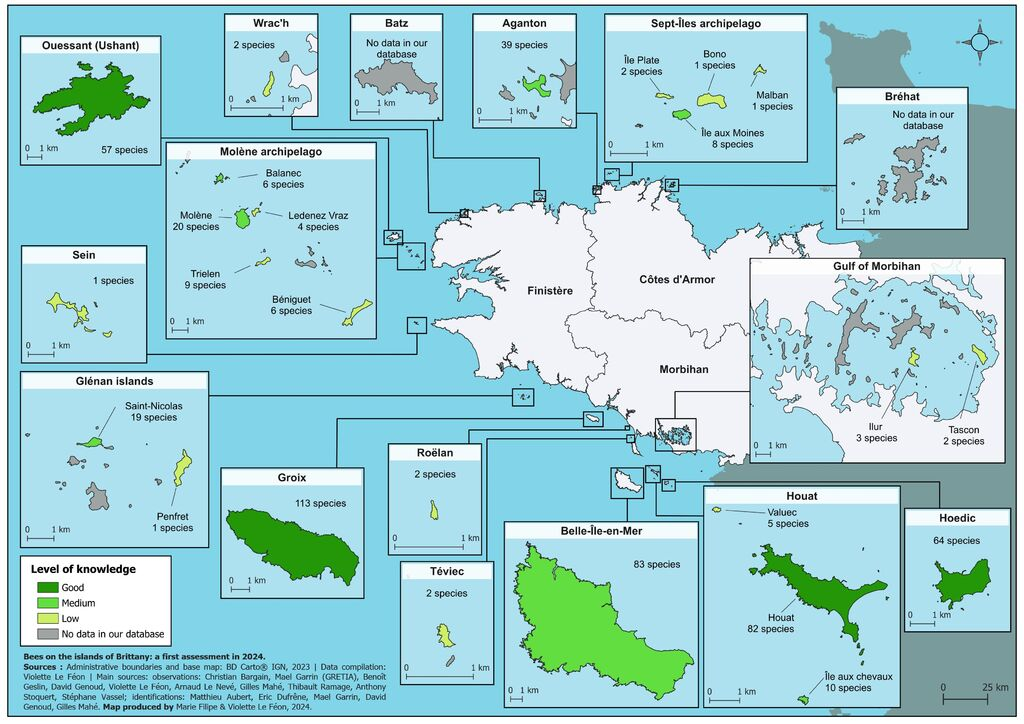
Map of the islands of Brittany providing the level of knowledge and the number of detected species. Islands represented in grey are those for which we have no data. We have shown the islands of Batz and Bréhat to highlight the lack of data for these two islands, which are part of the group of large islands in Brittany. The level of knowledge was assessed on the basis of the number of occurrence records we obtained. Three categories were defined: * low, ** medium and *** good level of knowledge. An occurrence record is here defined as the observation of a number of specimens of a given taxon at a given location on a given date.

We obtained data for 25 islands (Fig. 2, Table 2), located in three departments of Brittany (Côtes d'Armor, Finistère and Morbihan). The area of these islands ranges from 1.2 hectares for Malban to 8563 hectares for Belle-Île-en-Mer. Tascon (55 ha), Sein (58 ha), Molène (75 ha), Hoedic (209 ha), Houat (288 ha), Groix (1482 ha), Ouessant (1558 ha) and Belle-Île-en-Mer are inhabited islands. The other islands are small entities where human presence is only occasional today (e.g. tourism, boating). The distance to the mainland ranges from a few hundred metres for islands connected to the mainland at low tide (Aganton, Tascon and Wrac'h) to more than 15 kilometres (the maximum distances being 18 km for Ouessant and 16 km for Hoedic). Overall, the islands of the south coast are relatively well represented in the dataset, whereas the islands of the north or west coasts are less well known (except Ouessant). Batz (370 ha) and Bréhat (309 ha), in particular, are two large islands for which we did not obtain any data.

The observation years range from 2000 to 2024, but most of the data (nearly 90%) come from the 2015-2024 period.

### Level of knowledge

The quantity and quality of the data are extremely variable. The number of occurrence records per island ranges from one (Malban and Penfret) to 582 (Groix). Thus, for several islands, we obtained only very occasional data. In contrast, some islands have benefitted from intensive surveys, with data collected throughout the entire flight period and over several years and in different locations and habitat types.

The classification of islands in the three groups is as follows (Table 2):

- low level of knowledge: 14 islands with less than 10 occurrence records;

- medium level of knowledge: six islands with a number of occurrence records between 10 and 100;

- good level of knowledge: four islands with more than 100 occurrence records. Groix, Hoedic, Houat and Ouessant were classified in this group. In addition to this quantitative aspect, the data for these four islands come from surveys carried out in different areas of the island, representative of the diversity of habitats, over several years (Suppl. material 3) and covering the entire flight period.

#### The particular case of Belle-Île-en-Mer

For Belle-Île-en-Mer, the number of occurrence records is 191, which could have placed this island in the ‘good level of knowledge’ category. However, a close examination of the quality of the data led us to place it in the ‘medium’ category. Indeed, the number of specimens examined (n = 261) seems low considering the size of the island. In addition, the analysis shows a lack of observations in early spring and late summer and, therefore, a possible significant underestimation of the number of species.

### Species richness

A total of 188 bee species, belonging to 28 genera, were detected. They are classified by family as follows: 40 Andrenidae, 45 Apidae, 19 Colletidae, 55 Halictidae, 26 Megachilidae and 3 Melittidae (Suppl. material 4).

The number of species detected per island ranged from one (Malban, Bono, Penfret and Sein) to 113 (Groix). For 17 islands, the number of species is between one and 10. This can provide valuable information in a species-focused approach (e.g. geographical distribution of a given species), but these data are obviously not sufficient to provide a comprehensive overview of the composition of the bee community on these islands.

For the four islands with a level of knowledge considered to be good, the number of species is 113 for Groix, 82 for Houat, 64 for Hoedic and 57 for Ouessant. In Belle-Île-en-Mer, 83 species were found.

### Species composition

#### Status in the European Red List of bees

Two species are classified as vulnerable (i.e. threatened species) in the European Red List of bees (*Bombusmuscorum* and *Colletesfodiens*) and 12 as near threatened (*Andrenahattorfiana*, *Andrenaovatula*, *Colletessuccinctus*, *Epeoluscruciger*, *Halictusquadricinctus*, *Lasioglossumbrevicorne*, *Lasioglossumpygmaeum*, *Lasioglossumquadrinotatum*, *Lasioglossumsexnotatum*, *Lasioglossumxanthopus*, *Sphecodeshyalinatus* and *Sphecodesspinulosus*). The other species are classified as least concern (134 species), data deficient (34 species) or are not evaluated.

#### Rarity status at the regional or national level

For each species, we calculated the number of islands on which it occurs and listed in Table 3 the species that were found on the largest number of islands (i.e. found on between 7 and 13 islands). We distinguished two categories in this list: (1) species common on the islands that are also common on the mainland and (2) species common on the islands that are threatened, declining or with a localised distribution on the mainland.

The first group includes the following species: *Andrenaflavipes*, *Andrenanigroaenea*, *Anthophoraplumipes*, *Bombuspascuorum*, *Bombuspratorum*, *Bombusterrestris*, *Halictusscabiosae*, *Lasioglossumcalceatum*, *Lasioglossummorio*, *Lasioglossumpunctatissimum* and *Lasioglossumvillosulum*.

The second group includes: *Andrenaagilissima*, *Bombusmuscorum*, *Colletesfodiens*, *Lasioglossumsmeathmanellum* and *Megachilelagopoda*. Different situations can be distinguished.

**Figure 3.**
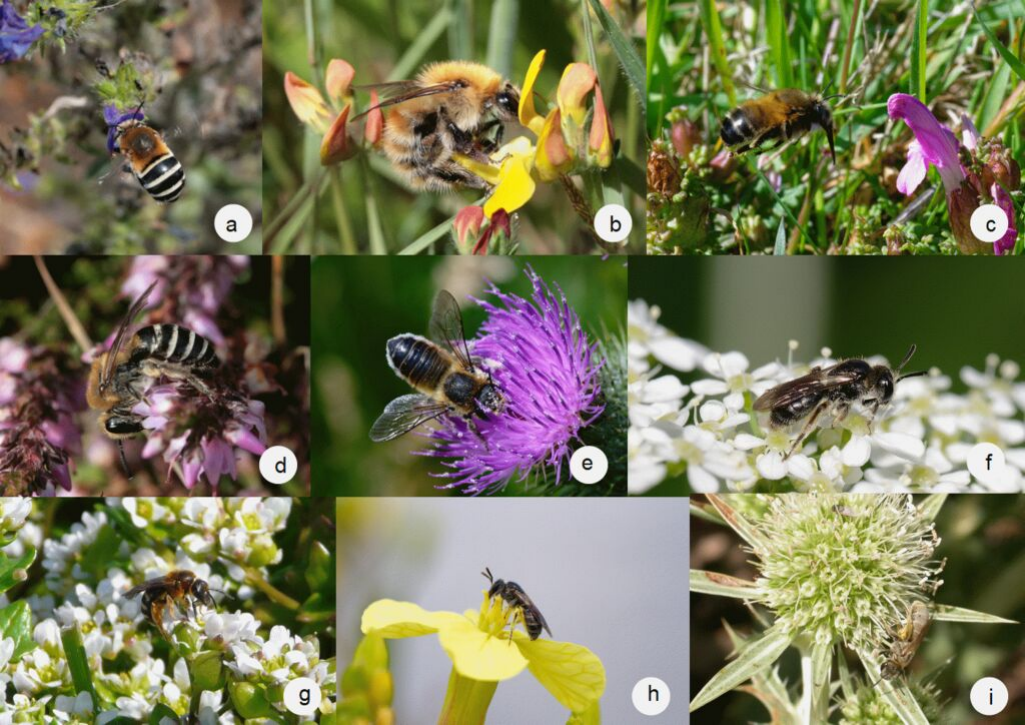
Some remarkable species recorded on the islands of Brittany: **a** *Amegillaquadrifasciata* (Belle-Île-en-Mer, 2017, photo: Mael Garrin); **b** *Bombushumilis* (Ouessant, 2017, photo: Mael Garrin); **c** *Anthophoramucida* (Groix, 2024, photo: David Genoud); **d** *Colletessuccinctus* (Aganton, 2018, photo: Mael Garrin); **e** *Megachilelagopoda* (Groix, 2024, photo: David Genoud); **f** *Andrenapusilla* (Groix, 2024, photo: David Genoud); **g** *Lasioglossumxanthopus* (Île aux Chevaux, 2016, photo: Anthony Stoquert); **h** *Lasioglossumnitidiusculum* (Groix, 2024, photo: David Genoud); **i** *Seladoniapollinosa* (Hoedic, 2008, photo: Arnaud Le Nevé).

- In Brittany, *Colletesfodiens* and *Andrenaagilissima* are associated with coastal habitats. They can be locally abundant, where their preferred flowers are abundant (respectively Asteraceae and Brassicaeae). While *A.agilissima* is classified as data deficient in the European Red List of bees (Nieto et al. 2014), *C.fodiens* is classified as vulnerable (i.e. a threatened species). Coastal areas of Brittany, such as those included in our study, could play a significant role for the conservation of this species at the European level.

- *Megachilelagopoda* (Fig. 3) is, after *Bombus* species and *Xylocopaviolacea*, one of the largest bee species listed in our study. Like *Halictusquadricinctus* (found in only two islands), *M.lagopoda* is dependent on high-quality habitats with abundant food resources.

- Finally, *Bombusmuscorum* seems to be the most remarkable species of Table 3. This species was found on four islands in the north-west (Balanec, Molène, Trielen and Ouessant), as well as on three islands in southern Brittany (Hoedic, Houat and Saint-Nicolas des Glénan). It was locally abundant: Devogel and Garrin (2022) found this species to be the dominant *Bombus* species on Balanec and Trielen (Molène archipelago). At the European level, it is a scarce and generally local bumblebee species which has declined in the last century (Else and Edwards 2018, Rasmont et al. 2021) and is assumed to have a low dispersal capability (Darvill et al. 2010). It is classified as vulnerable (i.e. a threatened species) in the European Red List of bees and a 'very high climate change risk' species in Rasmont et al. (2015) which means that climate change poses a very high risk to the species. Along with the Brière (an area of marshland to the east of Brittany, Mahé (2015)), several islands of Brittany could play a major role in its conservation on a regional and even national scale.

A large number of other species in our dataset are rare on a regional or even national scale and are also uncommon or rare on the studied islands (some of them are illustrated in Fig. 3): *Andrenahattorfiana*, *Andrenafuscipes*, *Andrenalabialis*, *Andrenapusilla*, *Andrenaschencki*, *Andrenasuerinensis*, *Panurgusbanksianus*, *Amegillaquadrifasciata*, *Ammobatespunctatus*, *Anthophoraaestivalis*, *Anthophoracrassipes*, *Anthophoramucida*, *Bombuscryptarum*, *Bombusjonellus*, *Bombushumilis*, *Bombusmagnus*, *Tetraloniaalticincta*, *Nomadarufipes*, *Colletessuccinctus*, *Halictusquadricinctus*, *Lasioglossumbrevicorne*, *Lasioglossumgriseolum*, *Lasioglossuminterruptum*, *Lasioglossumleucopus*, *Lasioglossumnigripes*, *Lasioglossumnitidiusculum*, *Lasioglossumquadrinotatum*, *Lasioglossumsemilucens*, *Lasioglossumxanthopus*, *Sphecodesalternatus*, *Sphecodescrassanus*, *Sphecodesspinulosus*, *Seladoniaconfusa*, *Seladoniapollinosa*, *Osmiagallarum*, *Coelioxyselongatus*, *Coelioxysinermis*, *Coelioxysmandibularis* and *Stelisbreviuscula*.

This list contains:

- some thermophilous species present on the islands of Morbihan (in particular Belle-Île-en-Mer, Hoedic and Houat) and which are not or only rarely found in the rest of the region (e.g. *Amegillaquadrifasciata*, *Anthophoramucida*, *Lasioglossumgriseolum*, *Seladoniapollinosa*, and *Sphecodesalternatus*);

- species associated with specific habitats. For example, on some islands, particularly Ouessant and Aganton, several species associated with heathland are found (*Andrenafuscipes*, *Bombuscryptarum*, *B.jonellus*, *B.humilis*, *B.magnus*, *Colletessuccinctus*, *Lasioglossumquadrinotatum* and *Nomadarufipes*);

- some cleptoparasitic species (see Sheffield et al. (2013) on the potential of cleptoparasitic bees as indicator taxa for assessing bee communities): *Ammobatespunctatus* (whose host is *Anthophorabimaculata*), *Nomadarufipes* (whose host is *Andrenafuscipes*; both species have been found only on Ouessant), *Sphecodesalternatus* and *S.crassanus* (hosts unconfirmed for these two species, according to Bogusch and Straka (2012)), *S.spinulosus* (whose host is *Lasioglossumxanthopus*), three species of *Coelioxys* (whose hosts are various species of *Megachile*) and *Stelisbreviuscula* (whose main host is *Heriadestruncorum*).

- species with specific feeding requirements (oligolectic species): for example, *Andrenahattorfiana* pollen specialist on Caprifoliaceae, *Andrenafuscipes* pollen specialist on Ericaceae, *Lasioglossumbrevicorne*, *Panurgusbanksianus* and *Tetraloniaalticincta* pollen specialists on Asteraceae and *Osmiagallarum* pollen specialist on Fabaceae.

## Discussion

The islands of Brittany have long played a major role in the conservation of flora, seabirds and marine mammals and their importance for conservation of both marine and continental invertebrates is increasingly recognised (e.g. GRETIA (2012), Buord et al. (2017), Ramage (2019)). In the past, there has been no assessment of the bee fauna on these islands and, until the mid-2010s, data on the occurrence of bees on these islands were very occasional. Since 2015, several studies have been launched, which have led to this initial assessment. By researching the data available from entomogists and in the databases of entomological associations, we gathered bee occurrence data for 25 continental islands in Brittany. These are located along the coastline of the region, on both the north and south coasts. They represent a wide variety of geographical (size and distance from the mainland), ecological (diversity of habitats) and climatic contexts.

A total of 188 bee species have been found, but this represents only an initial indication on the actual number of species. Other species are certainly still to be found, as we have no data for many islands, including two large ones, Batz and Bréhat. Moreover, the level of knowledge remains relatively poor for several islands. On Belle-Île-en-Mer in particular, it is certain that several species remain to be discovered. The habitats are diverse and of high quality, especially the grasslands (Laurent et al. 2022) and the first survey carried out a few years ago (Garrin 2018) showed that this island is likely to host species that are rare at the regional scale, in particular thermophilous species that usually have a more southerly distribution in France.

In general, the presence of a species on an island does not necessarily indicate the existence of an established and permanent population (see Beavis (2024) for the Isles of Scilly). In our study, nesting sites have been found on several islands, sometimes forming populous aggregations (e.g. *Anthophoraplumipes*, *Colletescunicularius*, *Colleteshederae*, *Lasioglossummalachurum*, *Panurgusbanksianus* and *Panurguscalcaratus*), suggesting the long-term establishment of populations. In most cases, however, the small number of individuals observed makes it difficult to draw conclusions. Some species may simply be represented by transient individuals, particularly on islands close to the mainland. Indeed, for large bees for example, individuals are known to travel significant distances and bumblebees are regularly observed flying over the sea (Livory 2009, Darvill et al. 2010, Livory 2011).

For some species on some islands, we only have relatively old observations (i.e. before 2010). This is the case for: *Bombuscryptarum* and *B.muscorum* on Ouessant, *B.campestris* and *B.veteranus* on Wrac'h, *B.magnus* on Groix and *Andrenamorio* on Houat. In some cases, specific, unsuccessful, surveys were carried out to look for the species on the islands where they were mentioned (*Bombuscryptarum* and *B.muscorum* on Ouessant and *B.magnus* on Groix). Their current presence should, therefore, have to be confirmed and, in the meantime, it is preferable to consider the data as ‘historical’.

Taken as a whole, the total species richness (188 species) shows that the islands of Brittany are a refuge for a large number of bee species. In France, more than 980 species have been recorded until now (Ropars et al. 2025), with the greatest richness found in southern France, in areas with a Mediterranean climate. In Brittany, current estimates indicate that there are more than 300 bee species (Observatoire des Abeilles 2018 and unpublished data). More than 62% of the known species in Brittany are, therefore, found on islands. In comparison, the species richness for Great Britain, Ireland and the Channel Islands is 275 (Falk and Lewington 2015). For the Channel Islands alone, the number of known species is around 170 (Ian Beavis, personal communication). The bees of this British archipelago, located to the north of Brittany, have been studied for much longer than those of the Breton islands and its fauna is certainly better known (Richards 1978, Beavis 1999, Else and Edwards 2018).

Considering the islands individually, comparisons are challenging due to significant differences in sampling effort and method. For four islands (Groix, Hoedic, Houat and Ouessant, Fig. 2), we consider that the level of knowledge is good. Several comments can be made. Firstly, despite their small size (288 and 209 ha, respectively) and their relatively large distance from the mainland (10 and 16 km, respectively), the islands of Houat and Hoedic are refuges for a relatively high number of species (82 and 64). Secondly, Groix, which is much larger (1482 ha) and located closer to the mainland (5 km), hosts 113 species i.e. more than 60% of the total species richness in our dataset. Interestingly, this species richness is similar to that found on the Mediterranean island of Porquerolles, which is 125 ha in size (Coiffait-Gombault et al. 2016). Thirdly, Groix and Ouessant are of the same size (1500 ha), but are located in contrasting climatic contexts. Although the inventories are certainly incomplete and could be improved, the difference in the number of species (113 vs. 57) could reflect a real difference between the two insular bee faunas, at least partly related to these climatic conditions. For example, some thermophilous species are present on Groix, but not on Ouessant, while several species associated with heathland have been found on Ouessant, but not on Groix.

**Figure 4.**
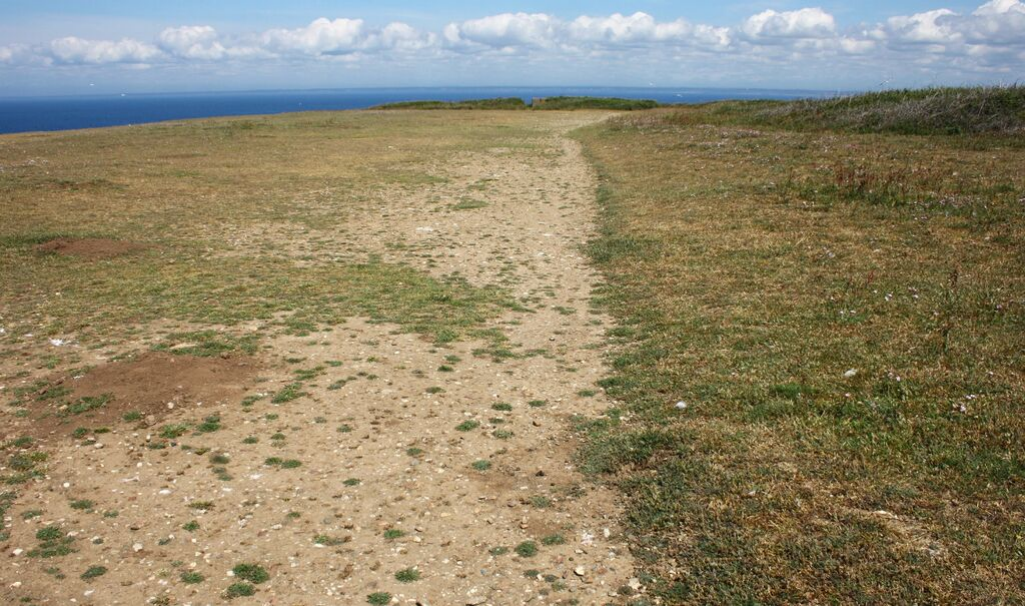
Cliff-top grassland at Pen Men, Groix. The bare track is used for nesting by several bee species: *Andrenaflavipes*, *Andrenanigroaenea*, *Colletescunicularius*, *Halictusscabiosae*, *Lasioglossummalachurum*, *Lasioglossummorio* and *Seladoniatumulorum*. The following cleptoparasitic species were also found: *Melectaalbifrons*, *Nomadalathburiana*, *Sphecodesmonilicornis* and *Sphecodespuncticeps* (photo: Violette Le Féon 2021).

**Figure 5.**
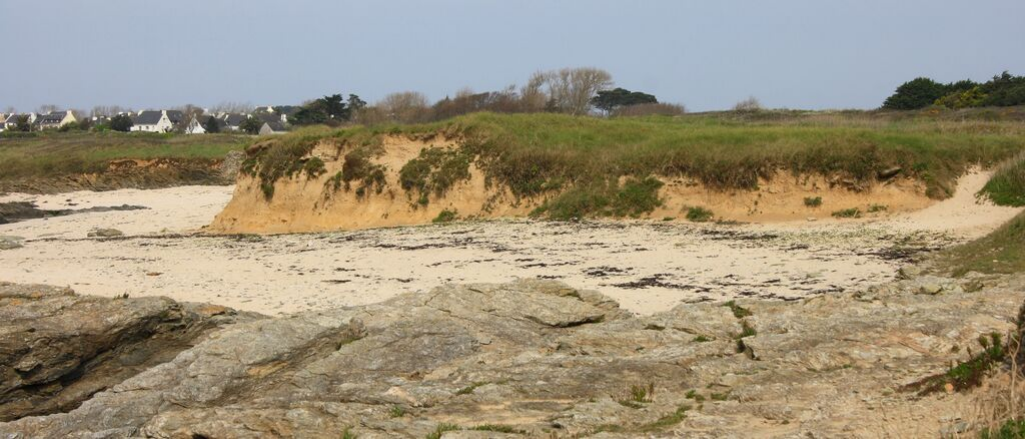
Soft rock cliff, covered in places with sea fennel (*Crithmummaritimum*), at Pointe des Chats, Groix. This south-facing slope is used for nesting by *Colletescunicularius*, *Halictusscabiosae*, *Lasioglossumleucopus*, *Lasioglossummalachurum*, *Lasioglossummorio* and *Lasioglossumvillosulum*. The following cleptoparasitic species were also found: *Nomadafabriciana*, *Nomadasuccincta*, *Sphecodesmonilicornis* and *Sphecodesniger* (photo: Violette Le Féon 2022).

**Figure 6.**
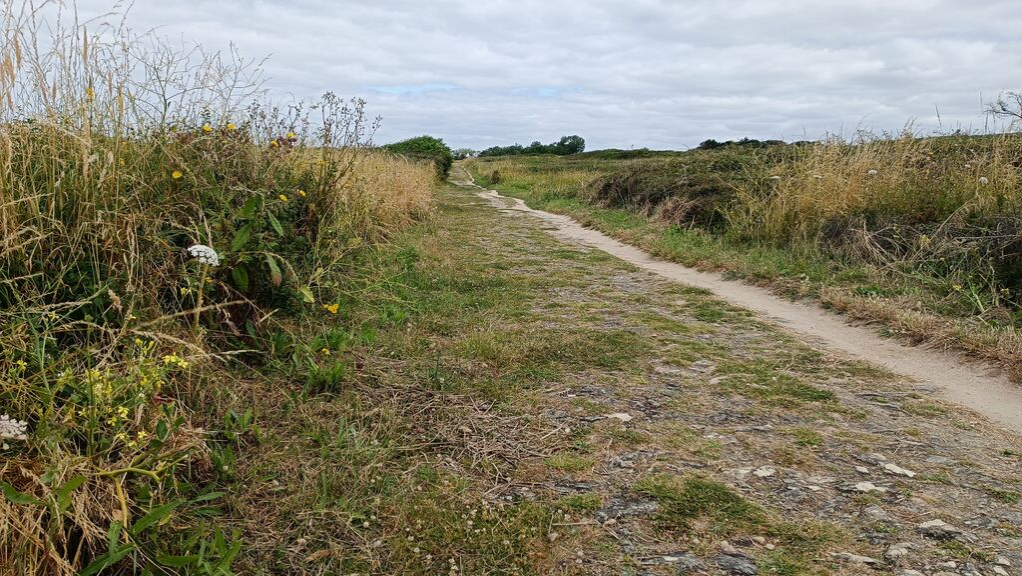
Pointe des Chats, Groix. The bare track is used for nesting by *Panurguscalcaratus* (photo: Violette Le Féon 2024).

**Figure 7.**
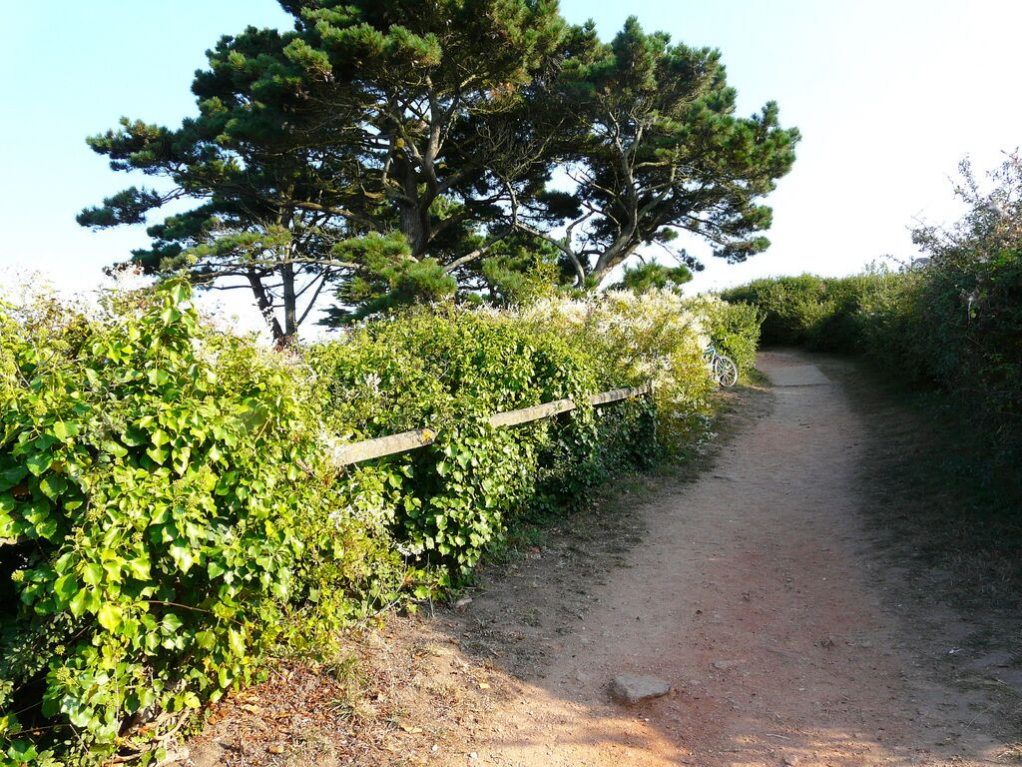
Port of Saint-Gildas, Houat. A nesting aggregation of *Colleteshederae* was observed in late summer 2016 (photo: Fanny Stoquert 2016).

**Figure 8.**
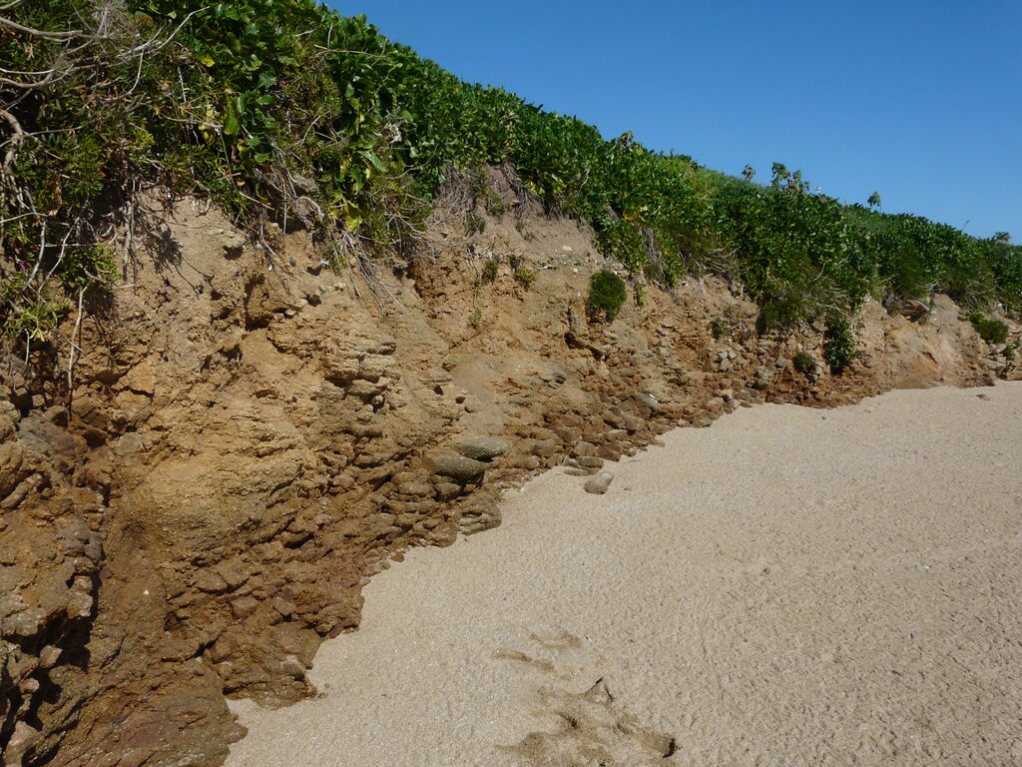
Soft rock cliff on the Île aux Chevaux, a small uninhabited island in the Houat-Hoedic archipelago. Several females of *Anthophoraplumipes* were observed nesting there in April 2016 (photo: Anthony Stoquert 2016).

Besides species richness, this study provides several interesting results regarding taxonomic composition. As expected (Blondel 1995), the bee fauna of the islands of Brittany includes several species that are common on the nearby mainland, but we have also shown that it includes many species that are rare on a regional or national scale. This is probably due to the favourable environmental conditions on the islands and the absence of, or less intensive, human activities than on the mainland. Agriculture is either absent or uses fewer pesticides and chemical fertilisers. Natural habitats are still largely present and include suitable nesting sites (see examples Figs 4, 5, 6, 7, 8). Especially in southern islands, climatic conditions create favourable conditions for bees, which are typically insects that thrive in dry, warm habitats (Orr et al. 2021). The same environmental conditions have a positive influence on the development of an abundant and diverse flora, which, in turn, favours the abundance and diversity of bees.

Most of the islands of Brittany benefit from a protected status (e.g. European Natura 2000 network, national or regional nature reserve, regional nature park and marine nature park). To a certain extent, this ensures that biodiversity and natural habitats are taken into account. However, conservation measures often focus mainly on flora and vertebrates (birds and mammals, in particular) and more rarely on insects (mainly on butterflies). It seems crucial to include bees more often when defining protected areas and planning habitat and vegetation management (Forister et al. 2019). These measures should ensure the presence of abundant and diverse floral resources and nesting sites for bees (Roulston and Goodell 2011, Antoine and Forrest 2021).

Bee conservation on islands requires a detailed understanding of habitat use, particularly in relation to nesting sites. These nesting sites, whether natural (e.g. soft rock cliffs, bare or sparsely vegetated ground, standing dead trees or dead branches) or associated with human activity (e.g. old stone walls), often cover very small areas. Their destruction can threaten entire bee populations, especially on islands where opportunities for recolonisation may be limited.

## Supplementary Material

FE71AA79-4798-53E8-99AF-5CA8CB89B233 10.3897/BDJ.13.e138570.suppl1 Supplementary material 1 Vegetation map of the islands of Brittany Data type Maps Brief description This file contains the vegetation maps of the islands considered in the text. They are based on the maps produced by the Conservatoire Botanique National de Brest (Sellin et al. 2021). File: oo_1216902.pdf https://binary.pensoft.net/file/1216902 Violette Le Féon, Mael Garrin, David Genoud, Matthieu Aubert, Éric Dufrêne, Marie Filipe, Floriane Flacher, Thibault Ramage, Anthony Stoquert, Stéphane Vassel, Benoît Geslin

B59A5EAC-73A0-58A9-88EF-C4DD3A618372 10.3897/BDJ.13.e138570.suppl2 Supplementary material 2 Bees on the islands of Brittany: occurrence database supporting the present publication Data type Occurrence data Brief description This table contains the bee occurrence data supporting the present publication. "Occurrence data" is here defined as the observation of a number of specimens of a given taxon at a given location on a given date.
The taxonomic reference system used is TAXREF version 17.0 (TAXREF 2024).
The column 'type of coordinates' indicates if the longitude and the latitude were given for the sampling location ('precise') or for the centroid of the island ('centroid') when the precise coordinates are unknown.
Names of the persons who collected the data or identified specimens are given as follows: 'first name' 'last name'. File: oo_1216899.xlsx https://binary.pensoft.net/file/1216899 Violette Le Féon, Mael Garrin, David Genoud, Matthieu Aubert, Éric Dufrêne, Marie Filipe, Floriane Flacher, Thibault Ramage, Anthony Stoquert, Stéphane Vassel, Benoît Geslin

21D7821C-79DC-52FC-B0F6-1FD3F2C987EB 10.3897/BDJ.13.e138570.suppl3 Supplementary material 3 Years of observation for each island Data type Table Brief description This file shows the years for which we have bee occurrence data for each island. A black box means that our database contains information for the year in question, a white box means that we have no data. File: oo_1216922.xlsx https://binary.pensoft.net/file/1216922 Violette Le Féon, Mael Garrin, David Genoud, Matthieu Aubert, Éric Dufrêne, Marie Filipe, Floriane Flacher, Thibault Ramage, Anthony Stoquert, Stéphane Vassel, Benoît Geslin

67D290B5-3EB9-592B-9C6A-1E2E94DDDD51 10.3897/BDJ.13.e138570.suppl4 Supplementary material 4 Presence-absence data of bee species on each island and status in the European Red List of bees Data type Presence-absence data Brief description This file provides presence-absence data of bee species on each island, classified by archipelago and geographical area (department). The taxonomic reference system used is TAXREF version 17.0 (TAXREF 2024). It also provides the status in the European Red List of bees (Nieto et al. 2014): DD = data deficient; LC = least concern; NT = near threatened; VU = vulnerable. NA (not available) means that the species is not assessed in the list. File: oo_1244833.xlsx https://binary.pensoft.net/file/1244833 Violette Le Féon, Mael Garrin, David Genoud, Matthieu Aubert, Éric Dufrêne, Marie Filipe, Floriane Flacher, Thibault Ramage, Anthony Stoquert, Stéphane Vassel, Benoît Geslin

## Figures and Tables

**Table 1.** References (articles or study reports) providing information on the acquisition framework for the data on bees on the islands of Brittany. Islands are classified by archipelago and geographical area (department). * For islands that are not associated with published references, we give the name of the person who collected the data and, in brackets, the name of the person who identified the bee specimens.

**Department**	**Archipelago**	**Island**	**References**
Côtes d’Armor	Sept-Îles	Île aux Moines	Le Viol et al. (2001), Courtial (2018)
		Bono	Le Viol et al. (2001)
		Malban	Courtial (2018)
		Île Plate	Courtial (2018)
	---	Aganton	Garrin (2020)
Finistère	Les Glénan	Penfret	Ramage (2017)
		Saint-Nicolas	Ramage (2017)
	Molène	Balanec	Devogel and Garrin (2022)
		Béniguet	Devogel and Garrin (2022)
		Trielen	Devogel and Garrin (2022)
		Ledenez Vraz	Garrin and Picard (2016), Garrin (2019)
		Molène	Garrin and Picard (2016), Garrin (2019)
	---	Wrac’h	*Gilles Mahé (Gilles Mahé, Pierre Rasmont)* *
	---	Ouessant	Kerbiriou et al. (2014), Garrin and Picard (2016), Garrin (2019)
	---	Sein	*Arnaud Le Nevé (Violette Le Féon)* *
Morbihan	Golfe du Morbihan	Tascon	*Yvan Brugerolles (Yvan Brugerolles)* *
		Ilur	*Ronan Arhuro (Ronan Arhuro)* *
	Houat-Hoedic	Hoedic	Le Féon et al. (2018)
		Houat	Le Féon et al. (2018)
		Île aux Chevaux	Le Féon et al. (2018)
		Valuec	Le Féon et al. (2018)
	---	Belle-Île-en-Mer	Garrin (2018)
	---	Groix	Le Féon (2023)
	---	Roëlan	Le Féon et al. (2018)
	---	Téviec	Le Féon et al. (2018)

**Table 2.** Number of occurrence records, number of specimens and species detected on each island, classified by archipelago and geographical area (department). The level of knowledge was assessed on the basis of the number of occurrence records we obtained. Three categories were defined: * low, ** medium and *** good level of knowledge. An occurrence record is here defined as the observation of a number of specimens of a given taxon at a given location on a given date.

**Department**	**Archipelago**	**Island**	**Area (ha)**	**Level of knowledge**	**Number of records**	**Number of specimens**	**Species richness**
Côtes d’Armor	Sept-Îles	Île aux Moines	9.4	**	20	76	8
		Bono	22	*	2	2	1
		Malban	1.2	*	1	1	1
		Île Plate	4.9	*	2	3	2
	---	Aganton	20	**	72	110	39
Finistère	Glénan	Penfret	39	*	1	1	1
		Saint-Nicolas	35	**	43	79	19
	Molène	Balanec	18	**	12	22	6
		Béniguet	63	*	7	21	6
		Trielen	27	*	9	24	9
		Ledenez Vraz	12	*	5	8	4
		Molène	75	**	22	43	20
	---	Wrac’h	4	*	2	2	2
	---	Ouessant	1558	***	318	794	57
	---	Sein	58	*	2	8	1
Morbihan	Gulf of Morbihan	Tascon	55	*	2	2	2
		Ilur	37	*	4	8	3
	Houat-Hoedic	Hoedic	209	***	201	686	64
		Houat	288	***	119	711	82
		Île aux Chevaux	3.5	**	10	61	10
		Valuec	3.4	*	5	13	5
	---	Belle-Île-en-Mer	8563	**	191	261	83
	---	Groix	1482	***	582	1689	113
	---	Roëlan	1.9	*	2	3	2
	---	Téviec	3.8	*	2	27	2

**Table 3.** Species found on the largest number of islands in our study (i.e. found on between 7 and 13 islands). The table provides the number of islands where these species have been found and their status in the European Red List (ERL) of bees (Nieto et al. 2014): DD = data deficient; LC = least concern; VU = vulnerable.

**Species**	**Number of islands where the species has been detected**	**Status in the ERL**
*Bombusterrestris*	13	LC
*Lasioglossummorio*	13	LC
*Colletesfodiens*	12	**VU**
*Lasioglossumsmeathmanellum*	12	LC
*Andrenaflavipes*	11	LC
*Andrenaagilissima*	10	DD
*Andrenanigroaenea*	9	LC
*Anthophoraplumipes*	8	LC
*Halictusscabiosae*	8	LC
*Lasioglossumcalceatum*	8	LC
*Lasioglossumpunctatissimum*	8	LC
*Bombusmuscorum*	7	**VU**
*Bombuspascuorum*	7	LC
*Bombuspratorum*	7	LC
*Lasioglossumvillosulum*	7	LC
*Megachilelagopoda*	7	LC
