# Supplementary material for: Exploring the bee fauna on the islands of Brittany (France): an initial survey reveals a remarkable species richness
Source: Biodivers Data J. 2025 Feb 14;13:e138570. doi: 10.3897/BDJ.13.e138570 (PMC11845982; doi:10.3897/BDJ.13.e138570)
Supplement: Supplementary material 1 — Vegetation map of the islands of Brittany [file bdj-13-e138570-s001.pdf]

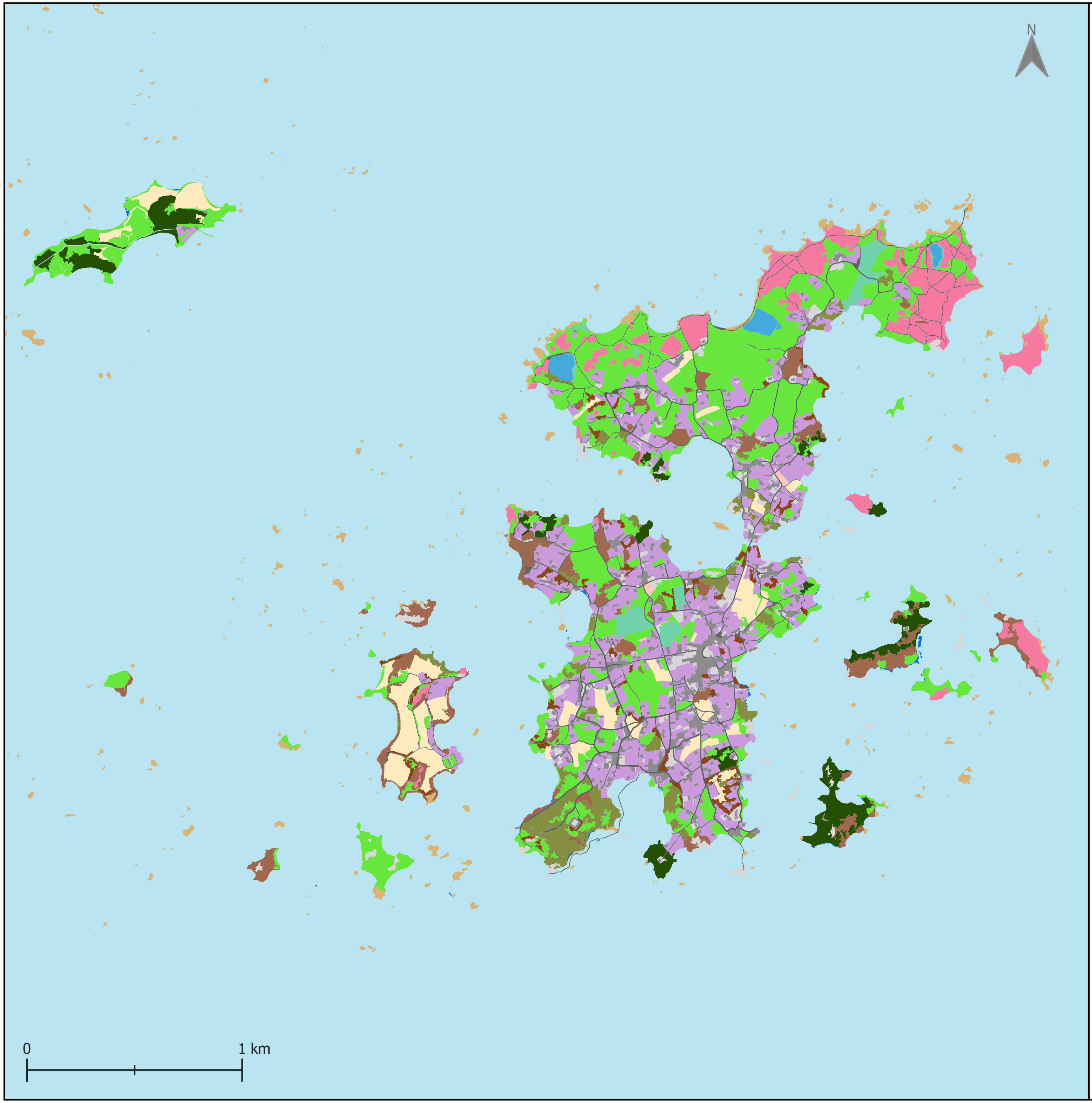

Bréhat

Natural and semi-natural vegetation

- Saltmarsh vegetations
- Dry and mesotrophic grasslands (excluding dunes)
- Wet grasslands (excluding salt marshes)
- Dry and mesic heaths
- Scrub and thickets
- Hedgerows
- Woodlands
- Vegetation of inland surface waters

Plantations, crops and gardens

- Coniferous plantations
- Orchards
- Arable lands
- Gardens

Areas with little or no terrestrial vegetation

- Marine habitats
- Buildings
- Roads
- Rock cliffs, sand beaches
- Other areas with little or no vegetation

Bees on the islands of Brittany: vegetation map of the island of Bréhat

Sources : Administrative boundaries and base - BD Carto® IGN, 2023 | Mapping the main vegetation types in Côtes d'Armor - CBN de Brest, 2019 ; Mapping the main vegetation types in Morbihan - CBN de Brest, 2020 ; Mapping the main vegetation types in Finistère - CBN de Brest, 2020

Map produced by Marie Filipe & Violette Le Féon, 2024

Sept-Îles archipelago

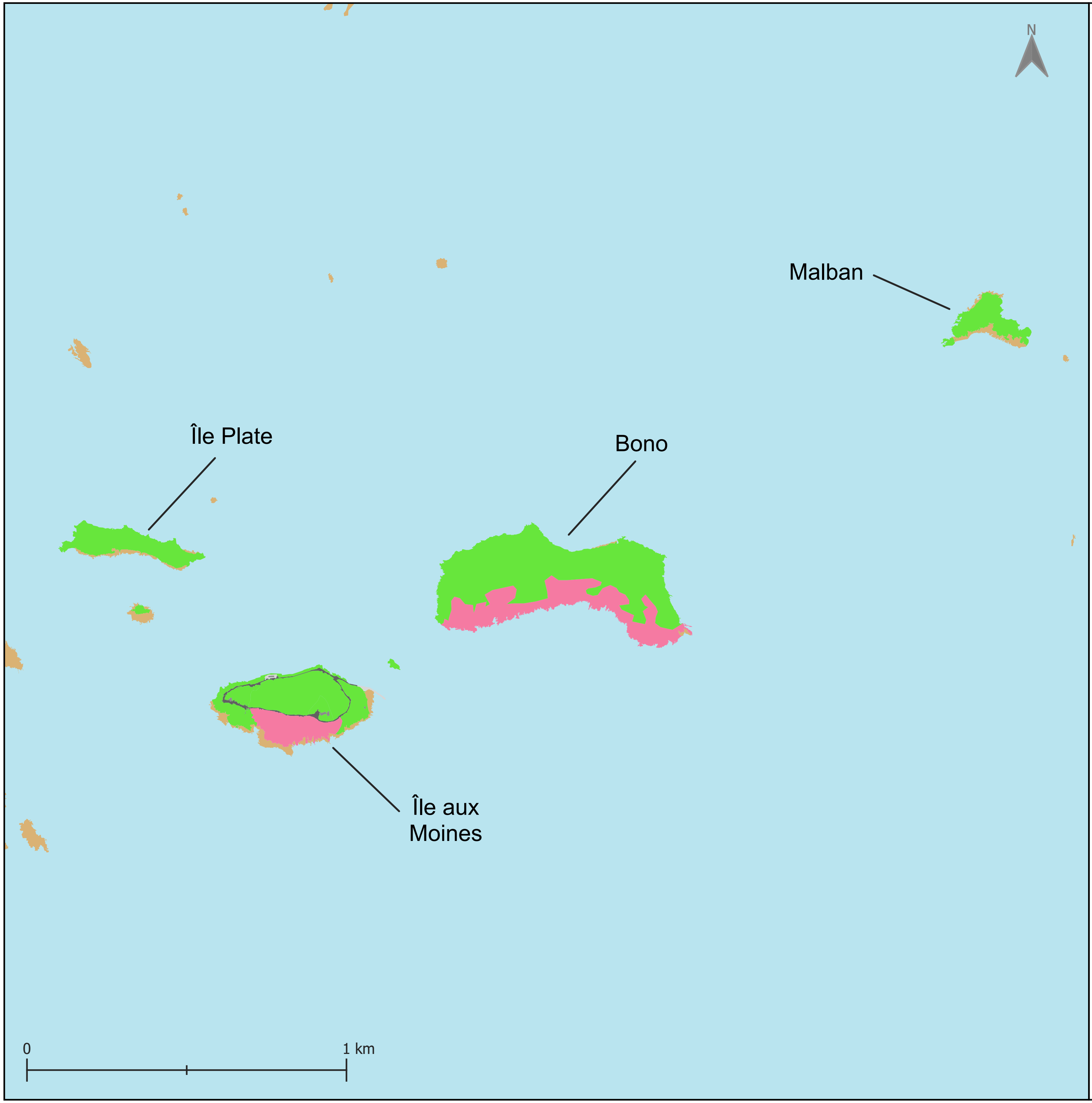

Natural and semi-natural vegetation

- Dry and mesotrophic grasslands (excluding dunes)
- Dry and mesic heaths

Areas with little or no terrestrial vegetation

- Marine habitats
- Buildings
- Roads
- Rock cliffs, sand beaches
- Other areas with little or no vegetation

Bees on the islands of Brittany: vegetation map of Sept-Îles archipelago

The map shows the islands for which we have bee data. The island of Rouzic is not shown.

Sources : Administrative boundaries and base - BD Carto® IGN, 2023 | Mapping the main vegetation types in Côtes d'Armor - CBN de Brest, 2019 ; Mapping the main vegetation types in Morbihan - CBN de Brest, 2020 ; Mapping the main vegetation types in Finistère - CBN de Brest, 2020

Map produced by Marie Filipe & Violette Le Féon, 2024

Aganton

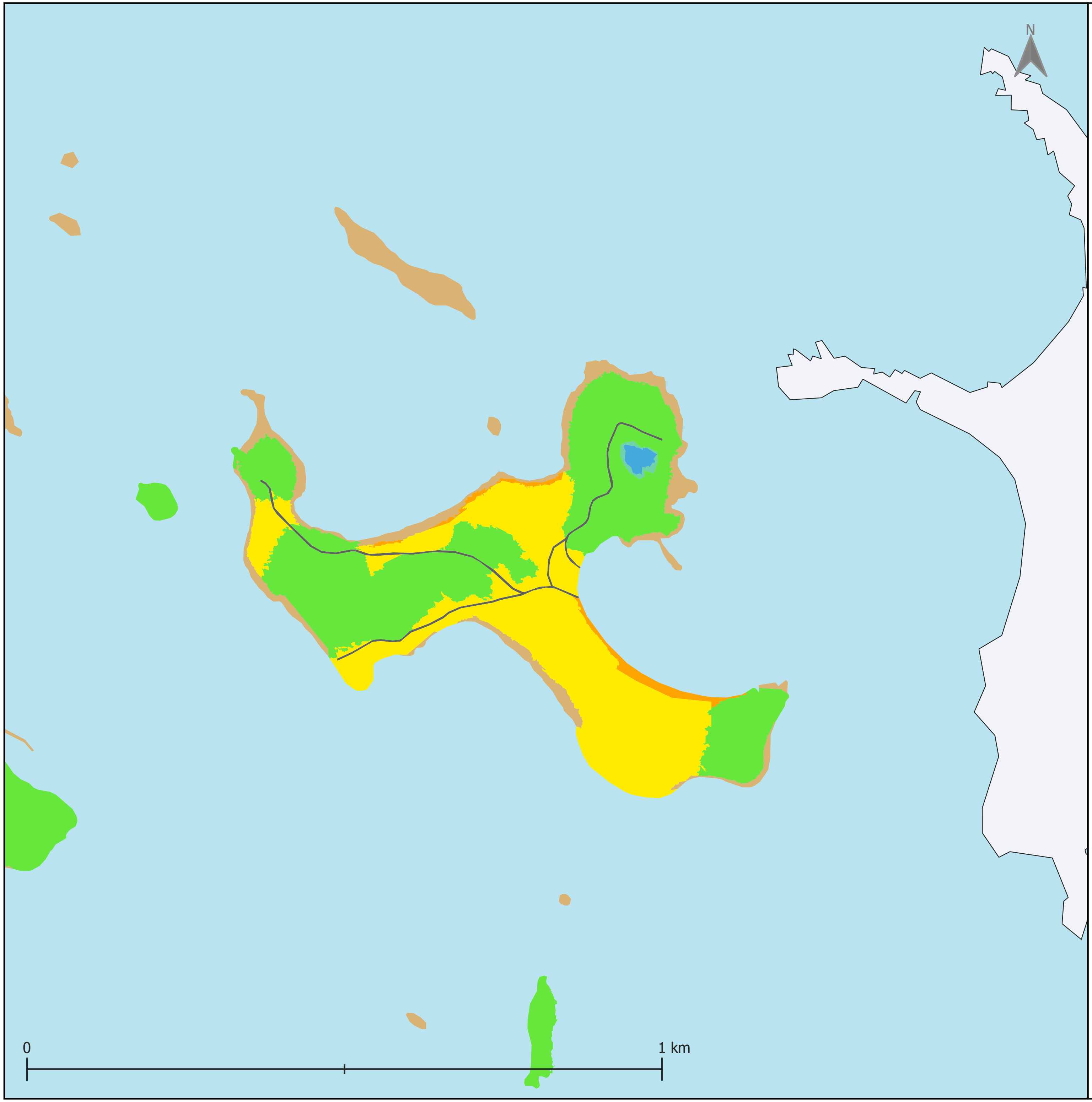

Natural and semi-natural vegetation

- Coastal stable dune grasslands (grey dunes)
- Shifting coastal dunes
- Dry and mesotrophic grasslands (excluding dunes)
- Wet grasslands (excluding salt marshes)
- Vegetation of inland surface waters

Areas with little or no terrestrial vegetation

- Marine habitats
- Roads
- Rock cliffs, sand beaches

Bees on the islands of Brittany: vegetation map of the island of Aganton

Sources : Administrative boundaries and base - BD Carto® IGN, 2023 | Mapping the main vegetation types in Côtes d'Armor - CBN de Brest, 2019 ; Mapping the main vegetation types in Morbihan - CBN de Brest, 2020 ; Mapping the main vegetation types in Finistère - CBN de Brest, 2020

Map produced by Marie Filipe & Violette Le Féon, 2024

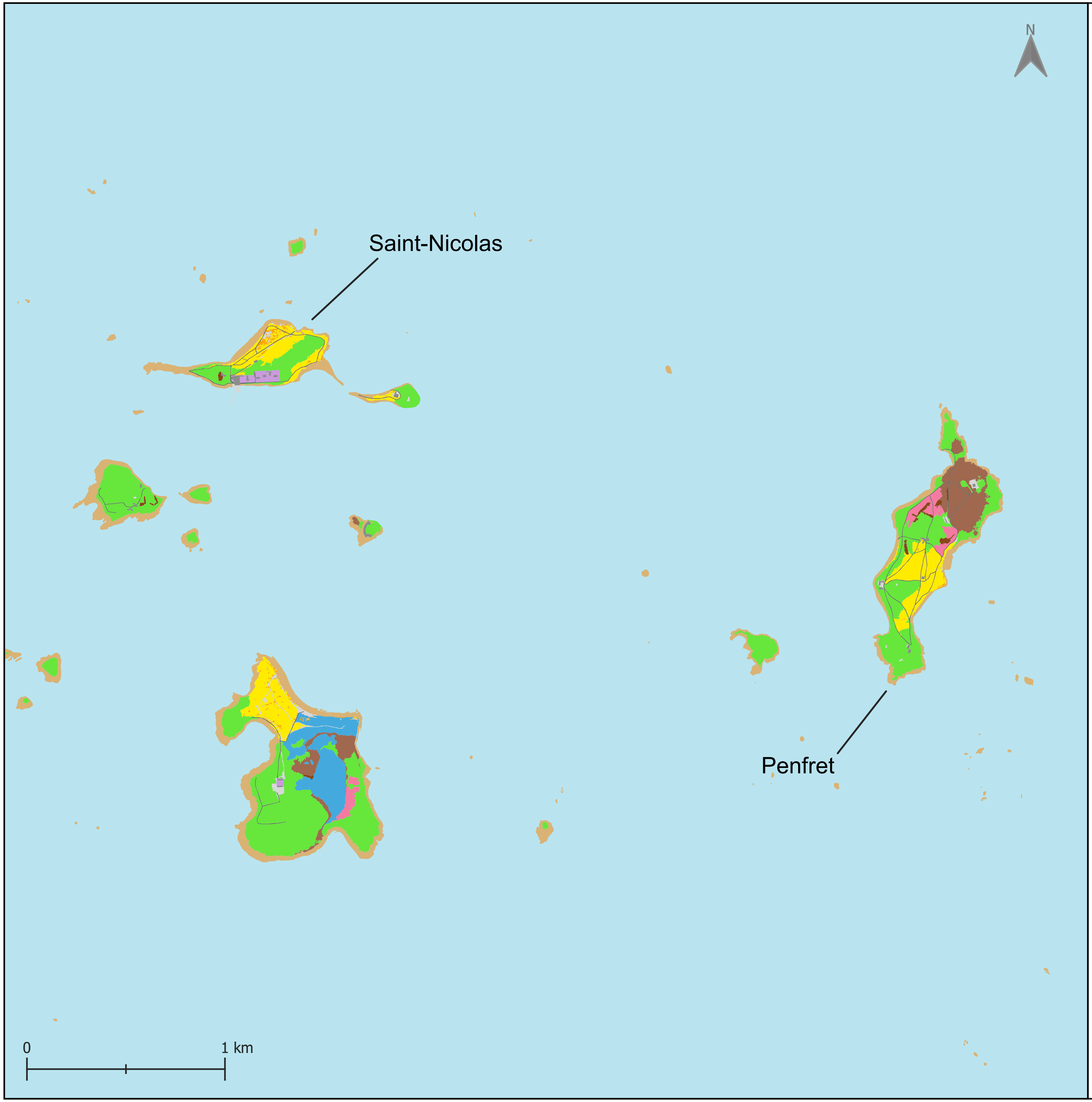

Glénan islands

Natural and semi-natural vegetation

- Coastal stable dune grasslands (grey dunes)
- Shifting coastal dunes
- Dry and mesotrophic grasslands (excluding dunes)
- Dry and mesic heaths
- Scrub and thickets
- Hedgerows
- Vegetation of inland surface waters

Plantations, crops and gardens

- Gardens

Areas with little or no terrestrial vegetation

- Marine habitats
- Buildings
- Roads
- Rock cliffs, sand beaches
- Other areas with little or no vegetation

Bees on the islands of Brittany: vegetation map of Glénan islands

The map shows the islands for which we have bee data. The "île aux Moutons" is not shown

Sources : Administrative boundaries and base - BD Carto® IGN, 2023 | Mapping the main vegetation types in Côtes d'Armor - CBN de Brest, 2019 ; Mapping the main vegetation types in Morbihan - CBN de Brest, 2020 ; Mapping the main vegetation types in Finistère - CBN de Brest, 2020

Map produced by Marie Filipe & Violette Le Féon, 2024

Molène archipelago

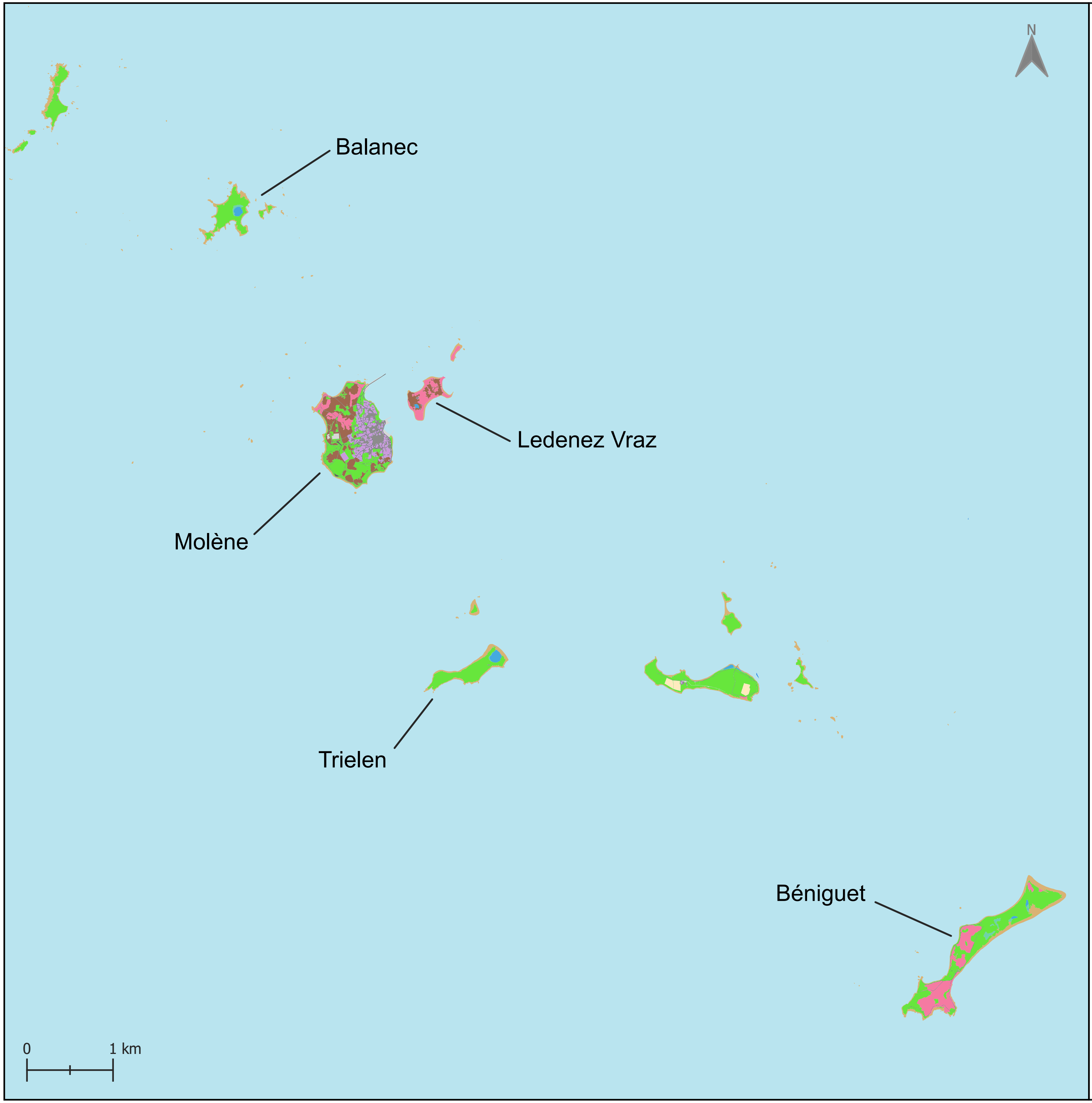

Natural and semi-natural vegetation

- Saltmarsh vegetations
- Dry and mesotrophic grasslands (excluding dunes)
- Wet grasslands (excluding salt marshes)
- Dry and mesic heaths
- Wet heaths
- Scrub and thickets
- Vegetation of inland surface waters

Plantations, crops and gardens

- Arable lands
- Gardens

Areas with little or no terrestrial vegetation

- Marine habitats
- Buildings
- Roads
- Rock cliffs, sand beaches
- Other areas with little or no vegetation

Bees on the islands of Brittany: vegetation map of Molène archipelago

Sources : Administrative boundaries and base - BD Carto® IGN, 2023 | Mapping the main vegetation types in Côtes d'Armor - CBN de Brest, 2019 ; Mapping the main vegetation types in Morbihan - CBN de Brest, 2020 ; Mapping the main vegetation types in Finistère - CBN de Brest, 2020

Map produced by Marie Filipe & Violette Le Féon, 2024

Wrac'h

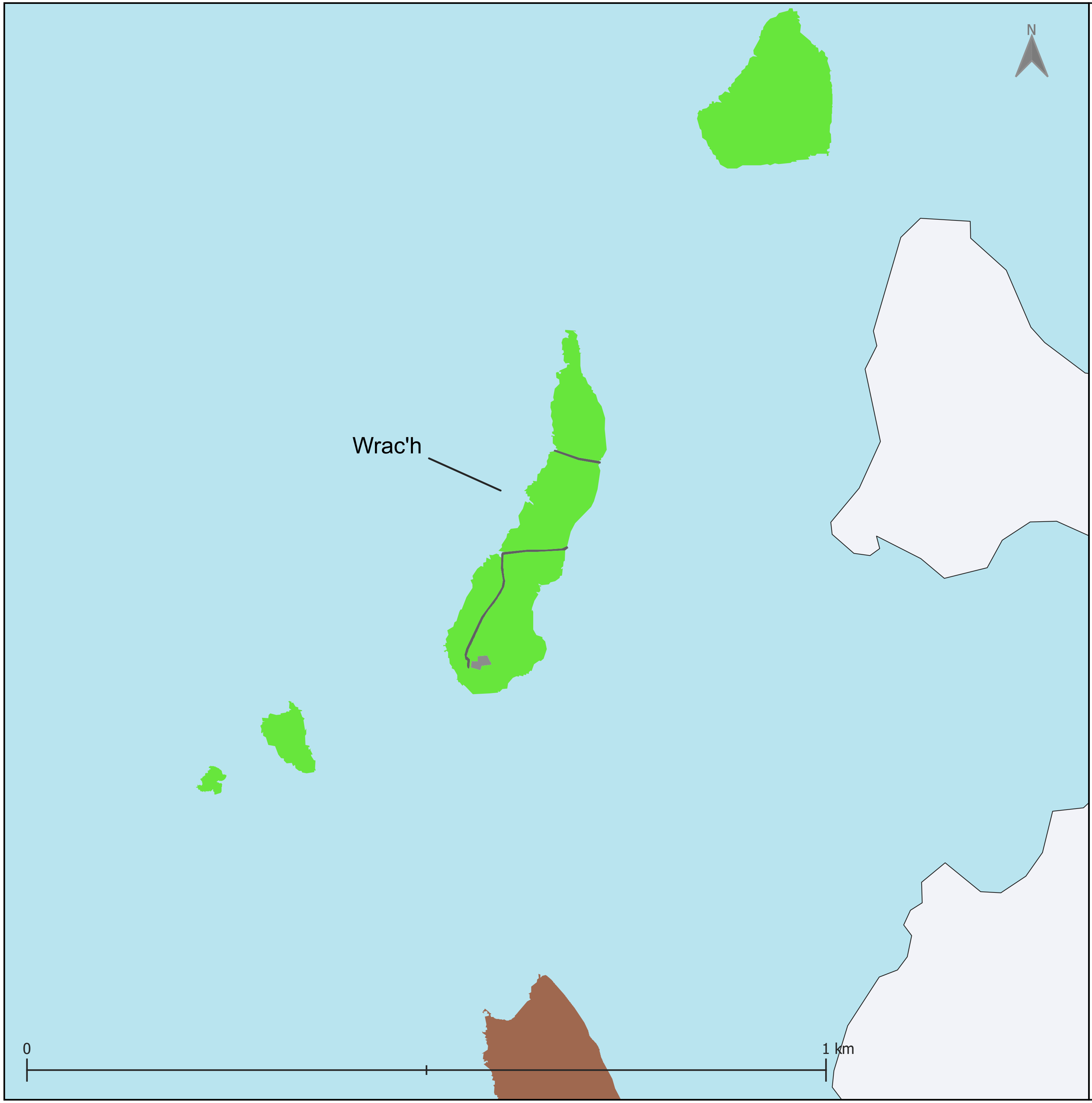

Natural and semi-natural vegetation

- Dry and mesotrophic grasslands (excluding dunes)
- Scrub and thickets

Areas with little or no terrestrial vegetation

- Marine habitats
- Buildings
- Roads

Bees on the islands of Brittany: vegetation map of the island of Wrac'h

Sources : Administrative boundaries and base - BD Carto® IGN, 2023 | Mapping the main vegetation types in Côtes d'Armor - CBN de Brest, 2019 ; Mapping the main vegetation types in Morbihan - CBN de Brest, 2020 ; Mapping the main vegetation types in Finistère - CBN de Brest, 2020

Map produced by Marie Filipe & Violette Le Féon, 2024

Ouessant (Ushant)

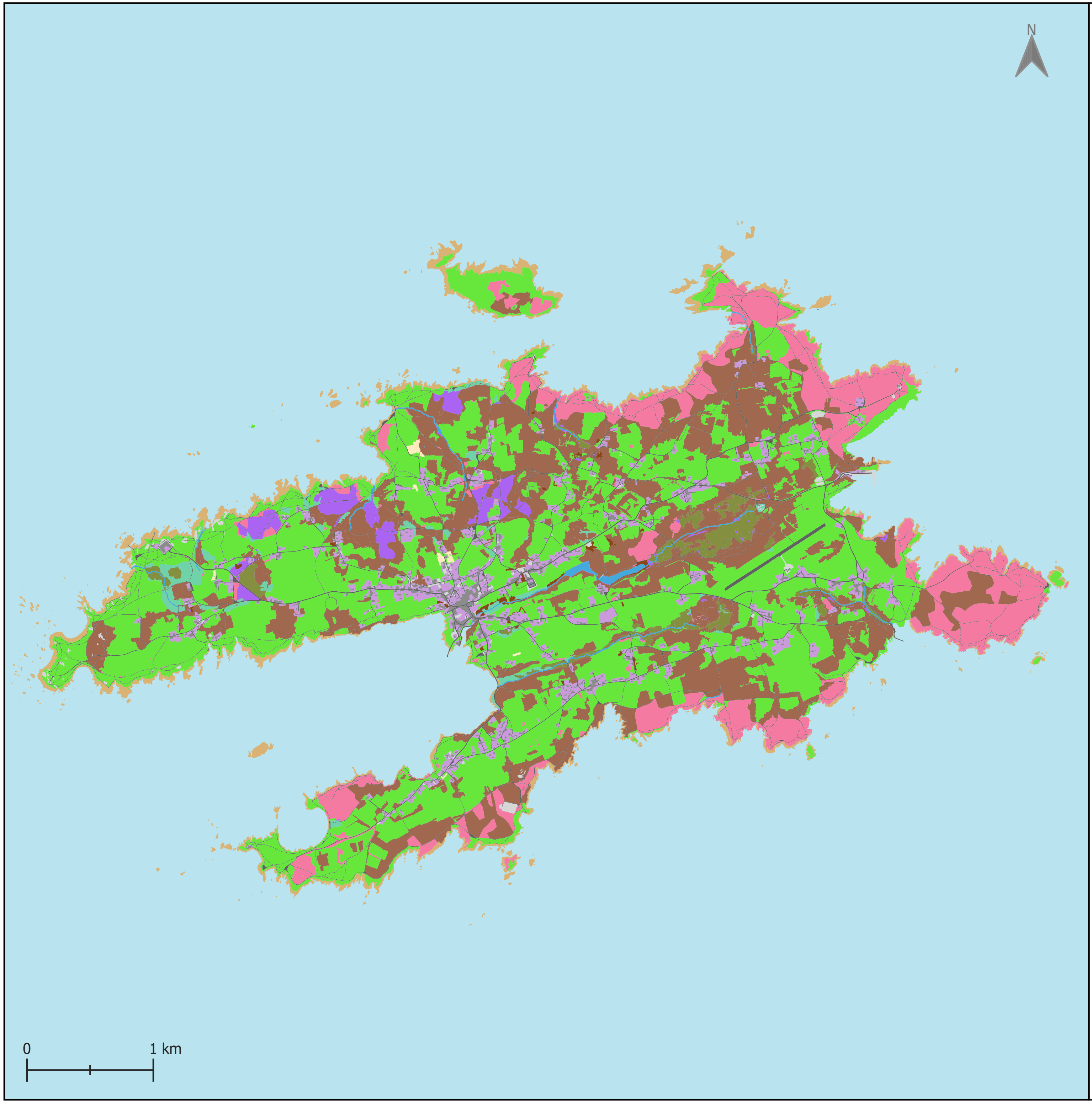

Natural and semi-natural vegetation

- Saltmarsh vegetations
- Dry and mesotrophic grasslands (excluding dunes)
- Wet grasslands (excluding salt marshes)
- Dry and mesic heaths
- Wet heaths
- Scrub and thickets
- Hedgerows
- Woodlands
- Vegetation of inland surface waters

Plantations, crops and gardens

- Arable lands
- Gardens

Areas with little or no terrestrial vegetation

- Marine habitats
- Buildings
- Roads
- Rock cliffs, sand beaches
- Other areas with little or no vegetation

Bees on the islands of Brittany: vegetation map of the island of Ushant

Sources : Administrative boundaries and base - BD Carto® IGN, 2023 | Mapping the main vegetation types in Côtes d'Armor - CBN de Brest, 2019 ; Mapping the main vegetation types in Morbihan - CBN de Brest, 2020 ; Mapping the main vegetation types in Finistère - CBN de Brest, 2020

Map produced by Marie Filipe & Violette Le Féon, 2024

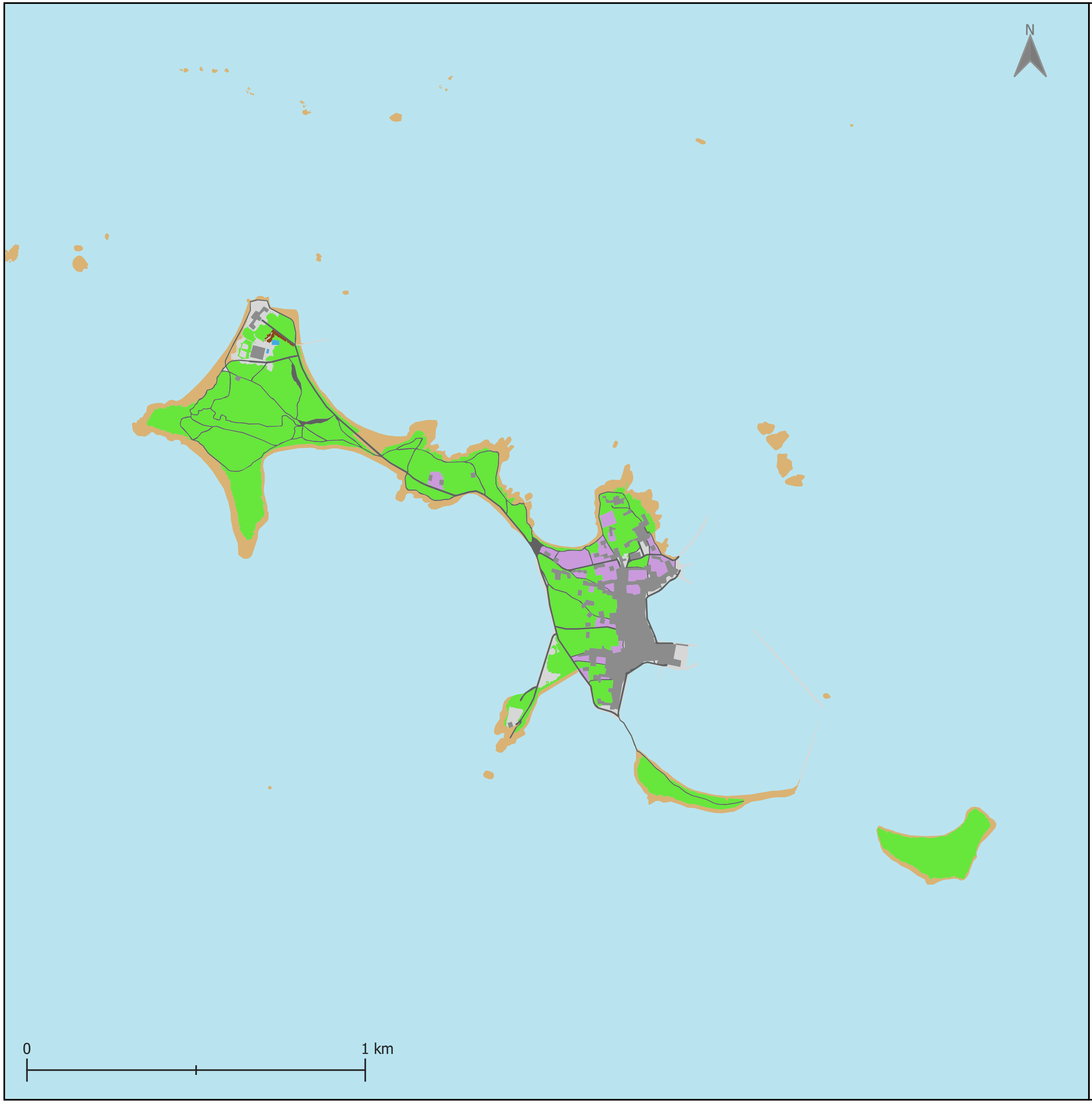

Sein

Natural and semi-natural vegetation

- Dry and mesotrophic grasslands (excluding dunes)
- Hedgerows
- Vegetation of inland surface waters

Plantations, crops and gardens

- Gardens

Areas with little or no terrestrial vegetation

- Marine habitats
- Buildings
- Roads
- Rock cliffs, sand beaches
- Other areas with little or no vegetation

Bees on the islands of Brittany: vegetation map of the island of Sein

Sources : Administrative boundaries and base - BD Carto® IGN, 2023 | Mapping the main vegetation types in Côtes d'Armor - CBN de Brest, 2019 ; Mapping the main vegetation types in Morbihan - CBN de Brest, 2020 ; Mapping the main vegetation types in Finistère - CBN de Brest, 2020

Map produced by Marie Filipe & Violette Le Féon, 2024

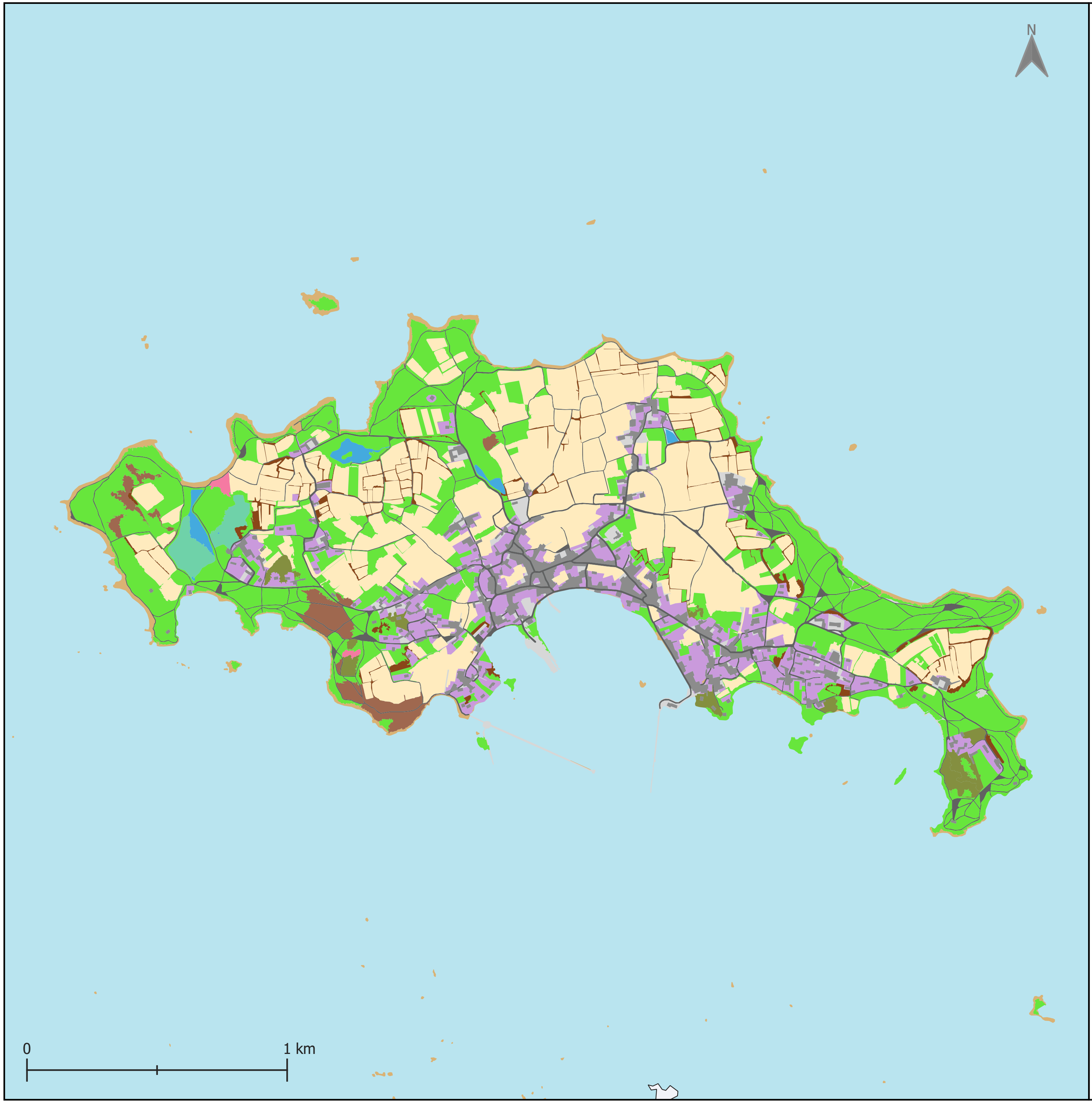

Batz

Natural and semi-natural vegetation

- Dry and mesotrophic grasslands (excluding dunes)
- Wet grasslands (excluding salt marshes)
- Dry and mesic heaths
- Scrub and thickets
- Hedgerows
- Woodlands
- Vegetation of inland surface waters

Plantations, crops and gardens

- Arable lands
- Gardens

Areas with little or no terrestrial vegetation

- Marine habitats
- Buildings
- Roads
- Rock cliffs, sand beaches
- Other areas with little or no vegetation

Bees on the islands of Brittany: vegetation map of the island of Batz

Sources : Administrative boundaries and base - BD Carto® IGN, 2023 | Mapping the main vegetation types in Côtes d'Armor - CBN de Brest, 2019 ; Mapping the main vegetation types in Morbihan - CBN de Brest, 2020 ; Mapping the main vegetation types in Finistère - CBN de Brest, 2020

Map produced by Marie Filipe & Violette Le Féon, 2024

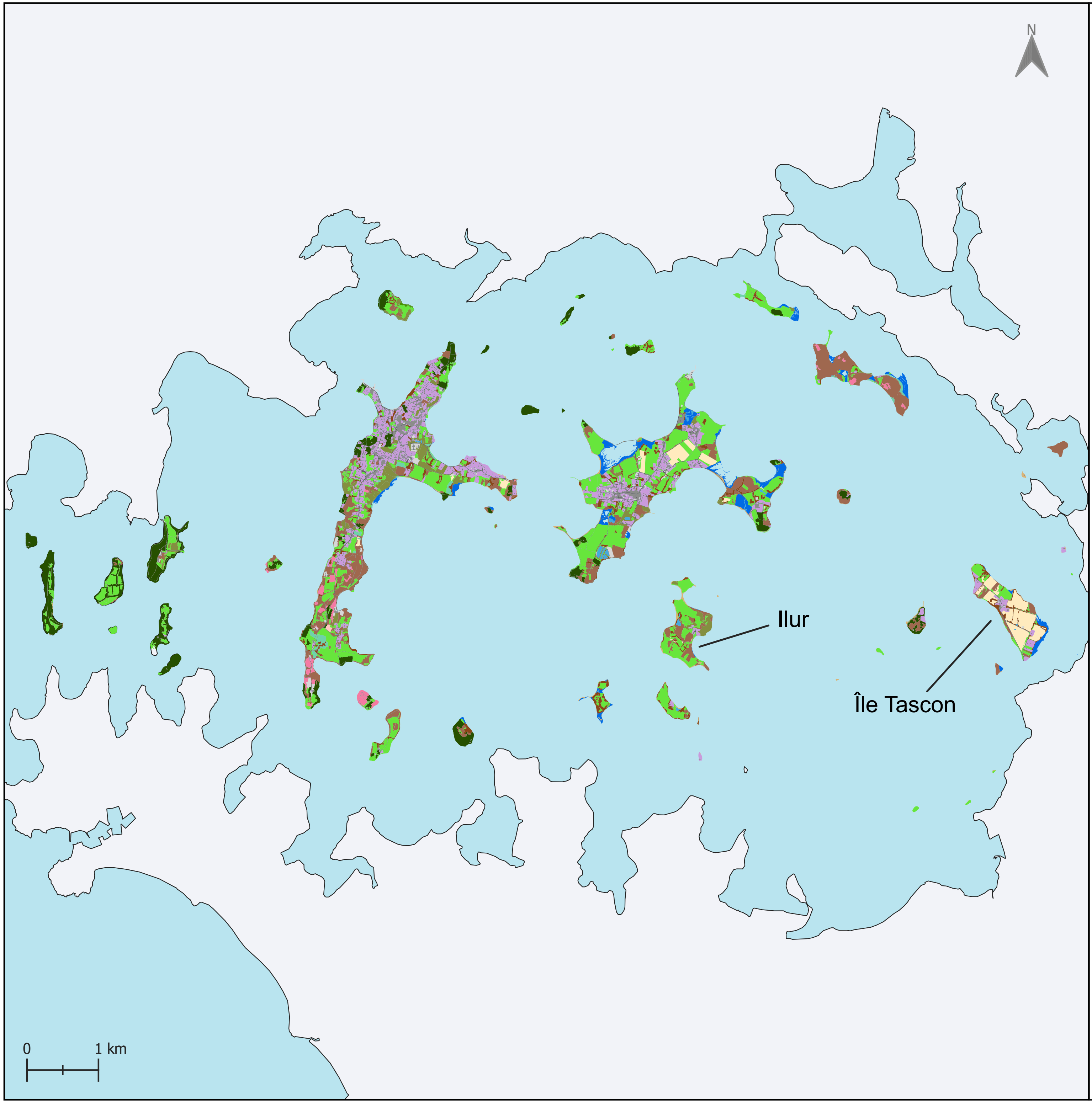

Gulf of Morbihan

Natural and semi-natural vegetation

- Saltmarsh vegetations
- Dry and mesotrophic grasslands (excluding dunes)
- Wet grasslands (excluding salt marshes)
- Dry and mesic heaths
- Wet heaths
- Scrub and thickets
- Hedgerows
- Woodlands
- Vegetation of inland surface waters

Plantations, crops and gardens

- Deciduous forestry plantations
- Coniferous plantations
- Orchards
- Arable lands
- Gardens

Areas with little or no terrestrial vegetation

- Marine habitats
- Buildings
- Roads
- Rock cliffs, sand beaches
- Other areas with little or no vegetation

Bees on the islands of Brittany: vegetation map in the Gulf of Morbihan

Sources : Administrative boundaries and base - BD Carto® IGN, 2023 | Mapping the main vegetation types in Côtes d'Armor - CBN de Brest, 2019 ; Mapping the main vegetation types in Morbihan - CBN de Brest, 2020 ; Mapping the main vegetation types in Finistère - CBN de Brest, 2020

Map produced by Marie Filipe & Violette Le Féon, 2024

Houat and surrounding islands

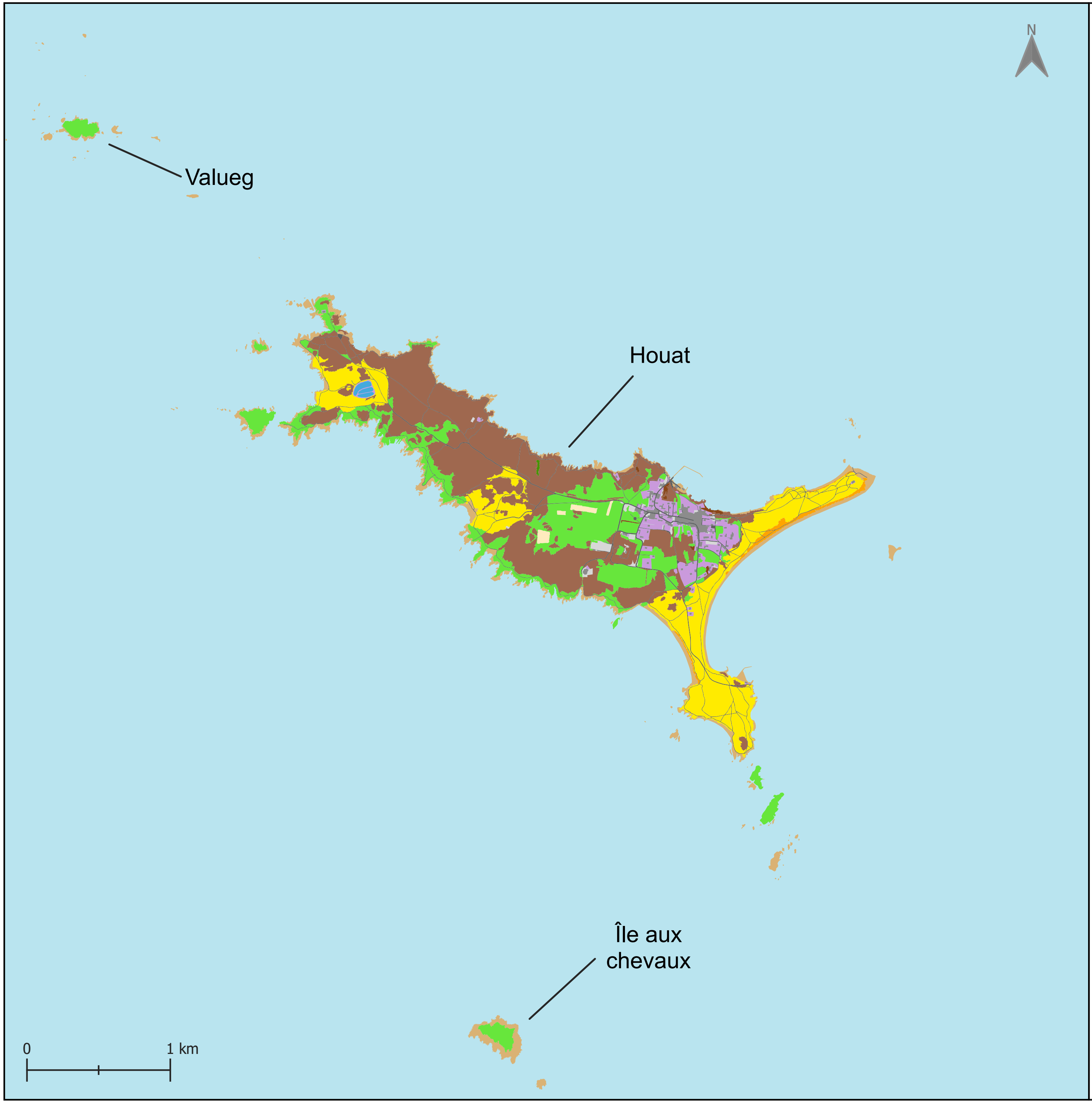

Natural and semi-natural vegetation

- Coastal stable dune grasslands (grey dunes)
- Shifting coastal dunes
- Dry and mesotrophic grasslands (excluding dunes)
- Scrub and thickets
- Hedgerows
- Vegetation of inland surface waters

Plantations, crops and gardens

- Deciduous forestry plantations
- Arable lands
- Gardens

Areas with little or no terrestrial vegetation

- Marine habitats
- Buildings
- Roads
- Rock cliffs, sand beaches
- Other areas with little or no vegetation

Bees on the islands of Brittany: vegetation map of the island of Houat and surrounding islands

Sources : Administrative boundaries and base - BD Carto® IGN, 2023 | Mapping the main vegetation types in Côtes d'Armor - CBN de Brest, 2019 ; Mapping the main vegetation types in Morbihan - CBN de Brest, 2020 ; Mapping the main vegetation types in Finistère - CBN de Brest, 2020

Map produced by Marie Filipe & Violette Le Féon, 2024

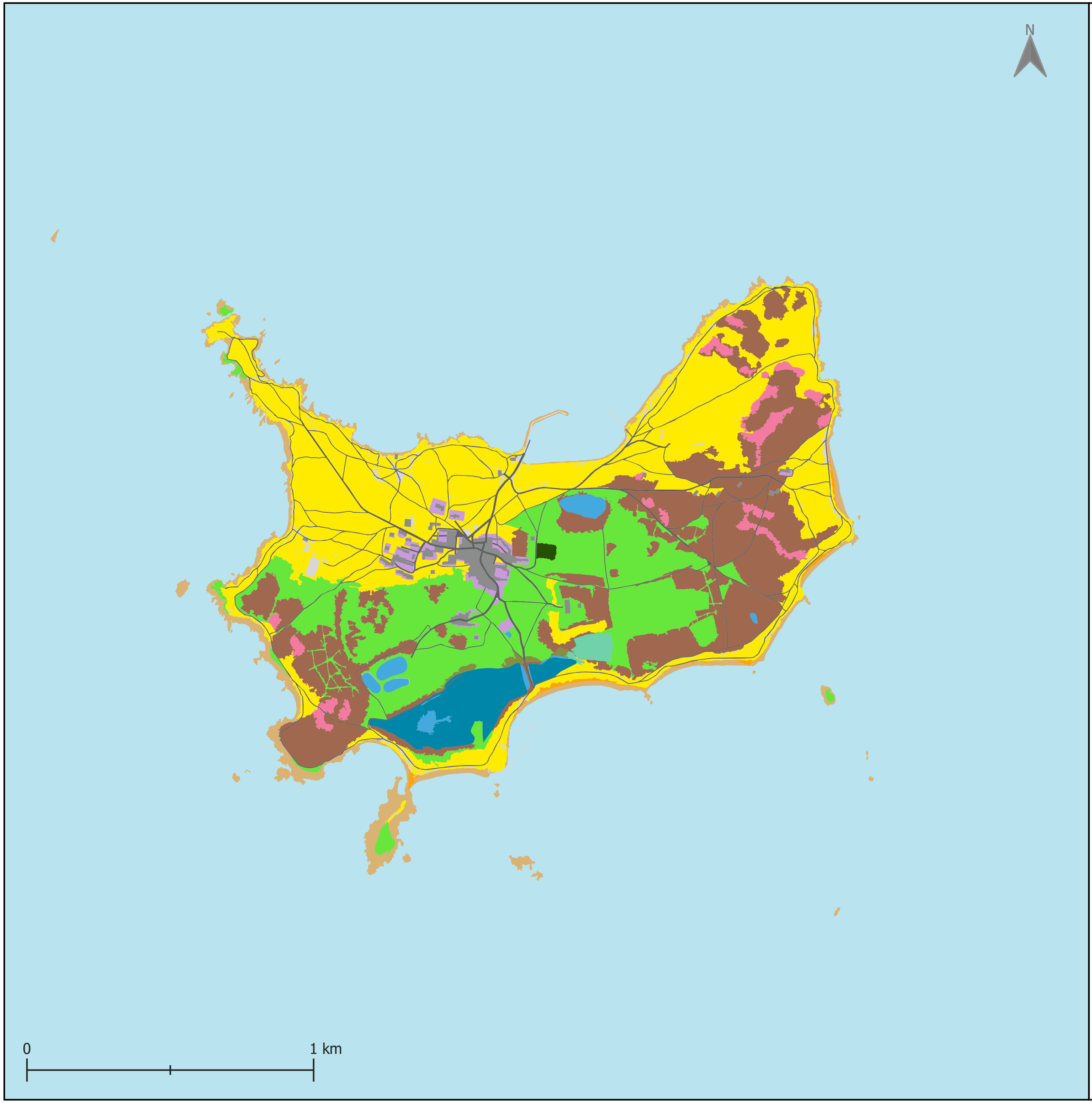

Hoedic

Natural and semi-natural vegetation

- Reedbeds
- Coastal stable dune grasslands (grey dunes)
- Shifting coastal dunes
- Dry and mesotrophic grasslands (excluding dunes)
- Wet grasslands (excluding salt marshes)
- Dry and mesic heaths
- Scrub and thickets
- Woodlands
- Vegetation of inland surface waters

Plantations, crops and gardens

- Coniferous plantations
- Gardens

Areas with little or no terrestrial vegetation

- Marine habitats
- Buildings
- Roads
- Rock cliffs, sand beaches
- Other areas with little or no vegetation

Bees on the islands of Brittany: vegetation map of the island of Hoedic

Sources : Administrative boundaries and base - BD Carto® IGN, 2023 | Mapping the main vegetation types in Côtes d'Armor - CBN de Brest, 2019 ; Mapping the main vegetation types in Morbihan - CBN de Brest, 2020 ; Mapping the main vegetation types in Finistère - CBN de Brest, 2020

Map produced by Marie Filipe & Violette Le Féon, 2024

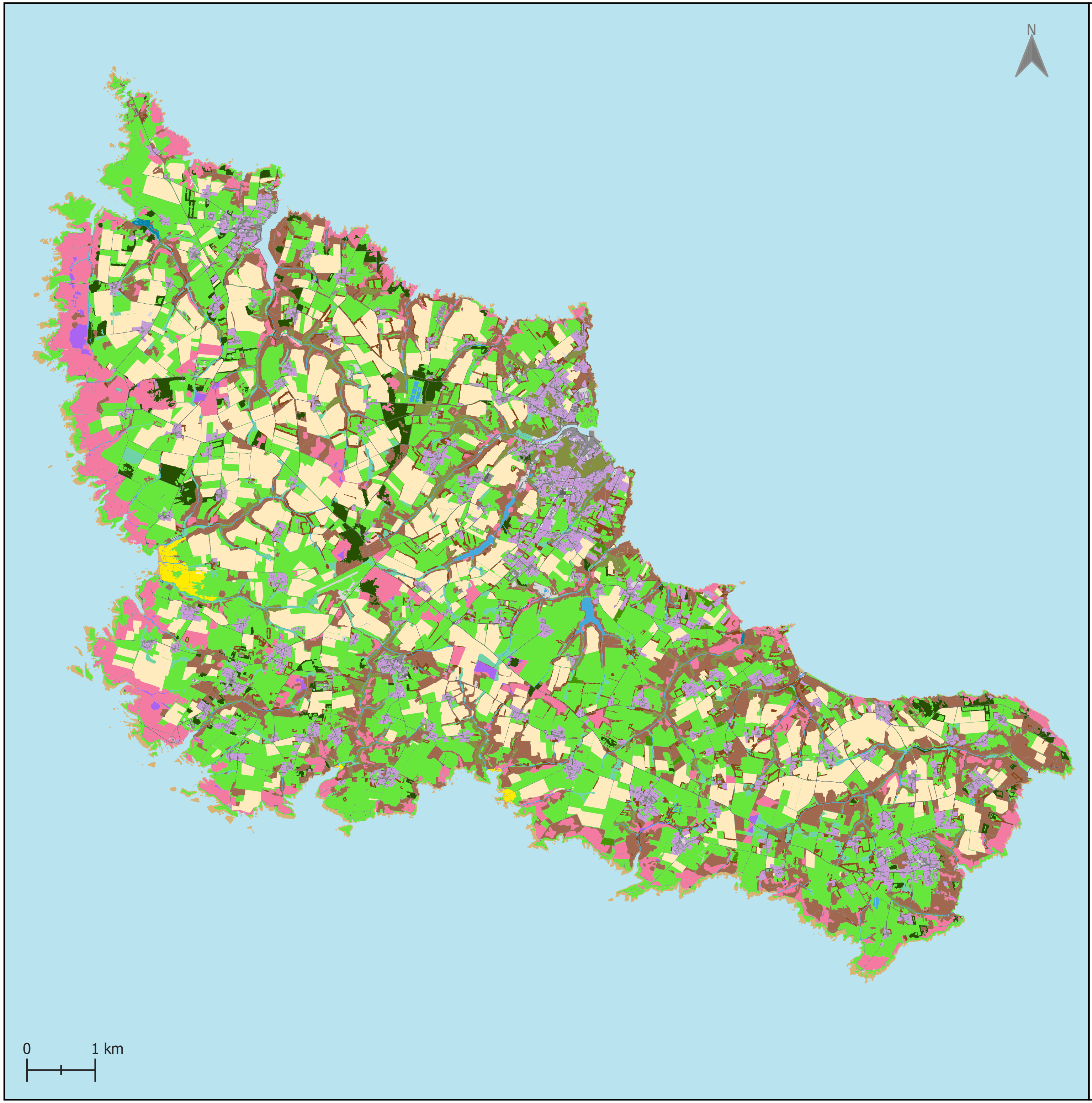

Belle-Île-en-Mer

Natural and semi-natural vegetation

- Reedbeds
- Coastal stable dune grasslands (grey dunes)
- Dry and mesotrophic grasslands (excluding dunes)
- Wet grasslands (excluding salt marshes)
- Dry and mesic heaths
- Wet heaths
- Scrub and thickets
- Hedgerows
- Woodlands
- Vegetation of inland surface waters

Plantations, crops and gardens

- Deciduous forestry plantations
- Coniferous plantations
- Orchards
- Arable lands
- Gardens

Areas with little or no terrestrial vegetation

- Marine habitats
- Buildings
- Roads
- Rock cliffs, sand beaches
- Other areas with little or no vegetation

Bees on the islands of Brittany: vegetation map of the island of Belle-Île-en-Mer

Sources : Administrative boundaries and base - BD Carto® IGN, 2023 | Mapping the main vegetation types in Côtes d'Armor - CBN de Brest, 2019 ; Mapping the main vegetation types in Morbihan - CBN de Brest, 2020 ; Mapping the main vegetation types in Finistère - CBN de Brest, 2020

Map produced by Marie Filipe & Violette Le Féon, 2024

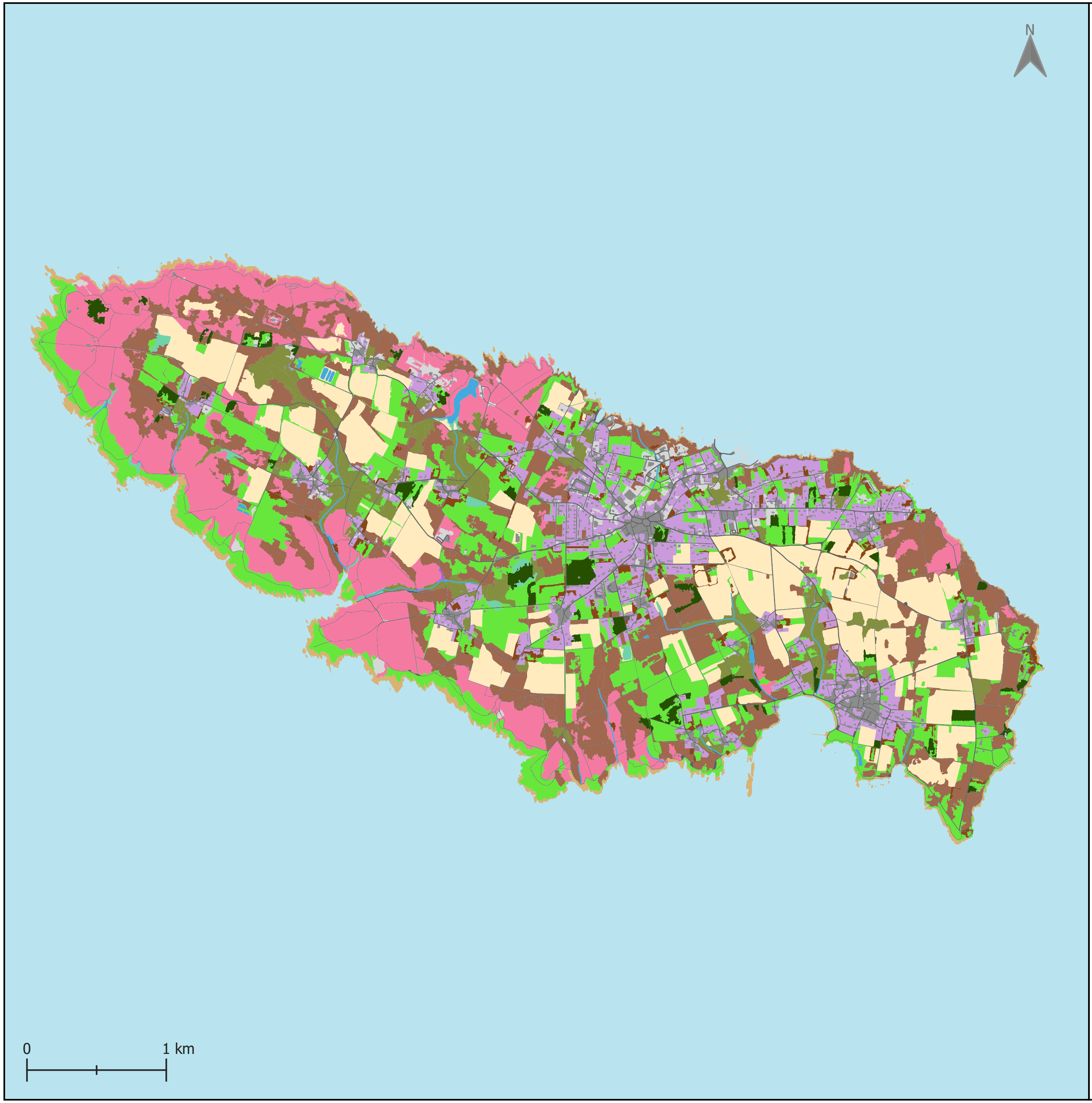

Groix

Natural and semi-natural vegetation

- Dry and mesotrophic grasslands (excluding dunes)
- Wet grasslands (excluding salt marshes)
- Dry and mesic heaths
- Wet heaths
- Scrub and thickets
- Hedgerows
- Woodlands
- Vegetation of inland surface waters

Plantations, crops and gardens

- Coniferous plantations
- Arable lands
- Gardens

Areas with little or no terrestrial vegetation

- Marine habitats
- Buildings
- Roads
- Rock cliffs, sand beaches
- Other areas with little or no vegetation

Bees on the islands of Brittany: vegetation map of the island of Groix

Sources : Administrative boundaries and base - BD Carto® IGN, 2023 | Mapping the main vegetation types in Côtes d'Armor - CBN de Brest, 2019 ; Mapping the main vegetation types in Morbihan - CBN de Brest, 2020 ; Mapping the main vegetation types in Finistère - CBN de Brest, 2020

Map produced by Marie Filipe & Violette Le Féon, 2024

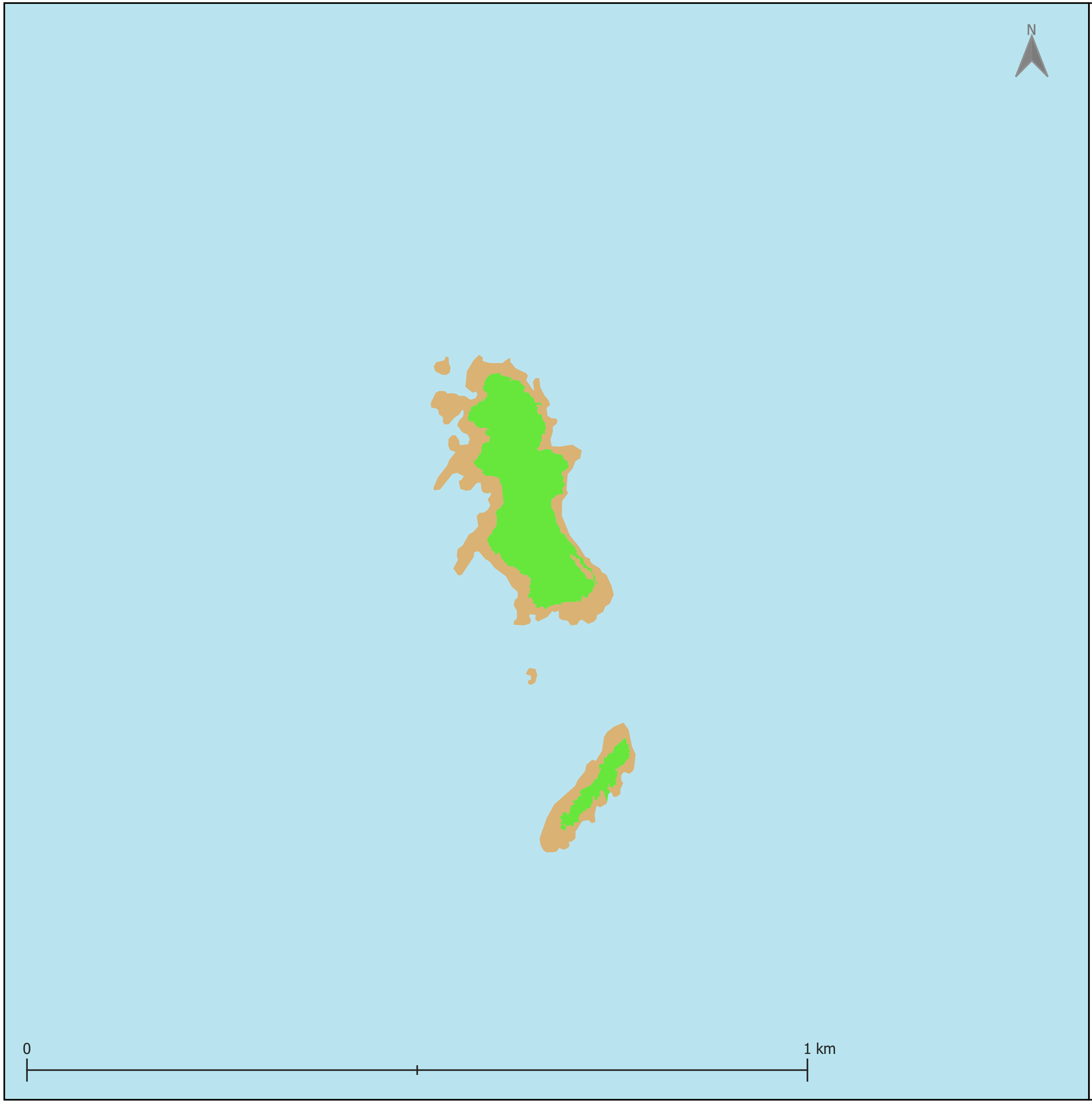

Téviec

Natural and semi-natural vegetation

Dry and mesotrophic grasslands (excluding dunes)

Areas with little or no terrestrial vegetation

Marine habitats

Rock cliffs, sand beaches

Bees on the islands of Brittany: vegetation map of the island of Téviec

Sources : Administrative boundaries and base - BD Carto® IGN, 2023 | Mapping the main vegetation types in Côtes d'Armor - CBN de Brest, 2019 ; Mapping the main vegetation types in Morbihan - CBN de Brest, 2020 ; Mapping the main vegetation types in Finistère - CBN de Brest, 2020

Map produced by Marie Filipe & Violette Le Féon, 2024

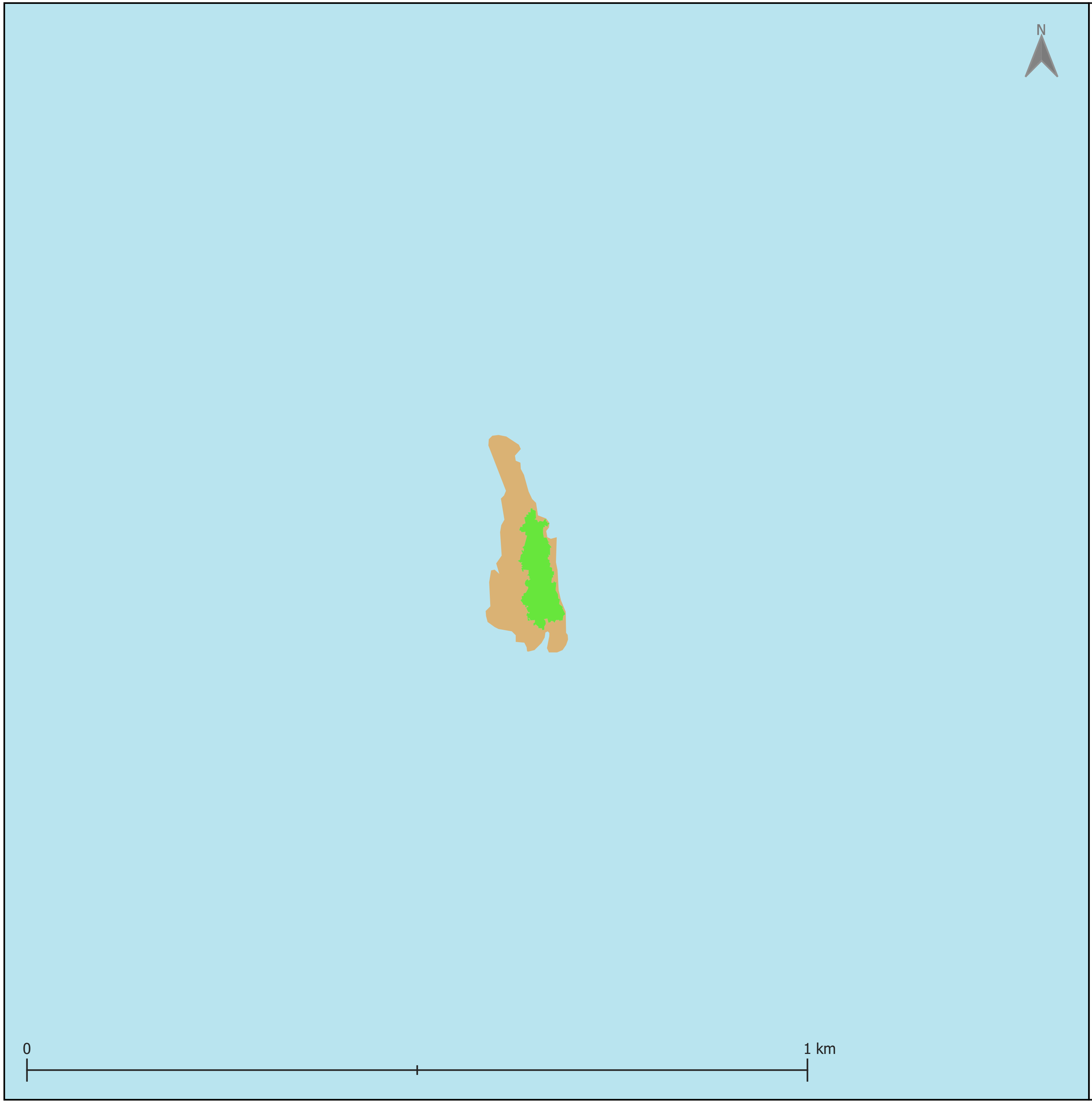

Roëlan

Natural and semi-natural vegetation

Dry and mesotrophic grasslands (excluding dunes)

Areas with little or no terrestrial vegetation

Marine habitats

Rock cliffs, sand beaches

Bees on the islands of Brittany: vegetation map of the island of Roëlan

Sources : Administrative boundaries and base - BD Carto® IGN, 2023 | Mapping the main vegetation types in Côtes d'Armor - CBN de Brest, 2019 ; Mapping the main vegetation types in Morbihan - CBN de Brest, 2020 ; Mapping the main vegetation types in Finistère - CBN de Brest, 2020

Map produced by Marie Filipe & Violette Le Féon, 2024
